# Characterizing the informativeness of pathogen genome sequence datasets about transmission between population groups

**DOI:** 10.1098/rspb.2025.2983

**Published:** 2026-03-18

**Authors:** Cécile Tran-Kiem, Amanda C. Perofsky, Justin Lessler, Trevor Bedford

**Affiliations:** 1 Vaccine and Infectious Disease Division, Fred Hutchinson Cancer Center, Seattle, WA, USA; 2 Fogarty International Center, National Institutes of Health, Bethesda, MD, USA; 3 Network Science Institute, Northeastern University, Boston, MA, USA; 4 Roux Institute, Northeastern University, Portland, ME, USA; 5 Department of Epidemiology, University of North Carolina at Chapel Hill, Chapel Hill, NC, USA; 6 Johns Hopkins Bloomberg School of Public Health, Baltimore, MD, USA; 7 Howard Hughes Medical Institute, Seattle, WA, USA

**Keywords:** Biological applications, health and disease and epidemiology, genomics, theoretical biology, infectious diseases, mathematical modelling, genome sequencing, phylogeography, genomic resolution, study design

## Abstract

Pathogen genome analysis helps characterize transmission between population groups. The information carried by pathogen sequences comes from the accumulation of mutations within their genomes; thus, the pace at which mutations accumulate should determine the granularity of transmission processes that pathogen sequences can characterize. Here, we investigate how the complex interplay between mutation, transmission, population mixing and sampling impacts study power. First, we develop a conceptual probabilistic framework to quantify the ability of pairs of sequences in capturing between-group transmission history. This allows us to comprehensively explore the space of possible phylogeographic analyses by explicitly considering the pace at which mutations accumulate and the pace at which between-group transmission events occur. Using this framework, we identify a pathogen-intrinsic limit in the mixing scale at which their sequence data remain informative, with faster mutating pathogens enabling finer spatial characterization. Second, we perform a simulation study exploring a range of assumptions regarding sequencing intensity. The sample size further imposes a limit on the characterization of between-group transmission processes. This work highlights inherent horizons of resolvability for population mixing processes that depend on the interaction between evolution, transmission, mixing and sampling. Such considerations are important for the design of pathogen genomic studies.

## 1. Introduction

Pathogen sequencing is an invaluable tool for studying disease transmission patterns [1]. Analysing pathogen genomes alongside metadata describing characteristics of infected hosts from which pathogens were isolated has helped characterize the transmission of pathogens between population groups of varying sizes. For example, haemagglutinin (HA) phylogenies have shed light on the intercontinental spread of seasonal influenza viruses [1], and identical sequence occurrence patterns have illuminated SARS-CoV-2 transmission between age groups [2].

It is generally acknowledged that the fundamental power of genomic epidemiological studies arises from the fast pace at which genetic variation is generated within pathogen genomes [3], relative to the pace at which pathogens transmit between hosts. When transmission and mutation events occur over similar time scales, pathogen genomes isolated from infected hosts indeed contain information about underlying epidemiological processes [4]. Thus, analysing such genomic data can help reconstruct transmission chains [5] or characterize population-level patterns of pathogen spread [1,2]. Prior work has explored the ability of pathogen genomes to reconstruct transmission chains [3,5], with overall resolution depending on the evolutionary rate, generation time, transmission intensity and sampling effort. However, we still lack clear methods to evaluate both the power and limits of pathogen genome sequences in quantifying transmission at the population level (phylogeographic inference). Making such capabilities and limits explicit is important to guide study design, set realistic expectations about genomic epidemiology’s role for epidemic response and ensure the efficient use of sequencing resources.

**Figure 1.**
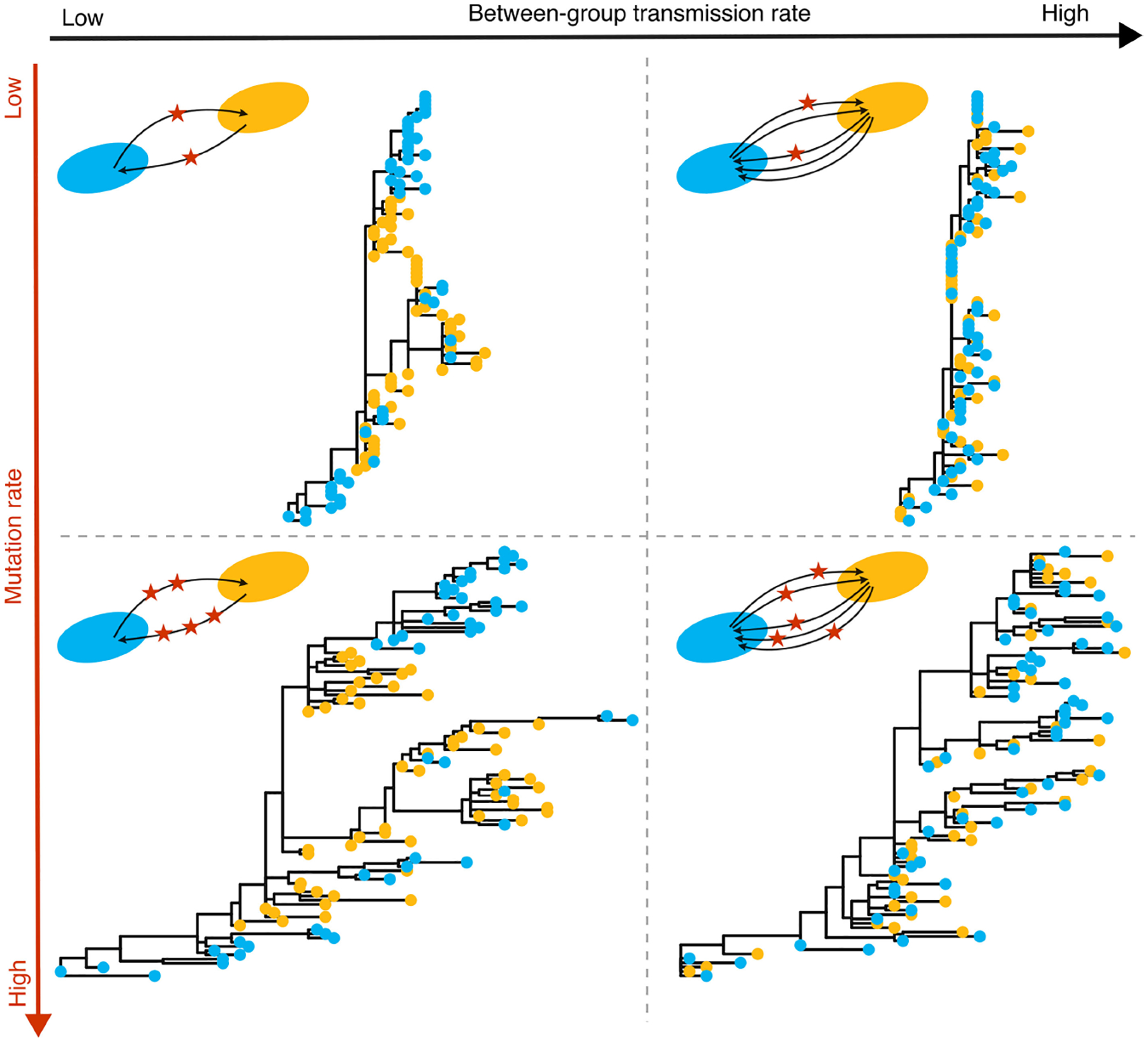
The ability of pathogen sequence data to characterize between-group transmission is impacted by the pace at which both between-group transmission and mutation events occur. To generate these figures, we simulate sequence data under an SEIR epidemic spreading between two groups of size 1000 (yellow and blue), assuming a genome length of 3000 bp. We assume a basic reproduction number of 1.5, that the mean time spent in the exposed compartment is 3 days, and that the mean time spent in the infectious compartment is 3 days. A total of 10% of infections are sequenced. We simulate the evolutionary process for a low mutation rate scenario (2 × 10^−5^ mutations per bp per day) and a high mutation rate scenario (8 × 10^−5^ mutations per bp per day). For the mixing process, infected individuals have a 98% probability of transmitting to someone within their group in the low between-group transmission scenario and a 50% probability in the high between-group transmission scenario. For each scenario, we include on the top left a toy figure to illustrate the frequency of between-group transmission events (number of arrows) and mutation events (number of stars).

To study such population processes, we expect the relative time scale at which mutation [6] and between-group transmission events occur to be critical [7]. This is because we anticipate pathogen genomes to be informative about a process only up to the rate at which novel genomic variation is observed [6,7]. If mutations accumulate at a much slower pace than between-group transmission events occur (high between-group transmission/low mutation rate in figure 1), genome sequences are insufficient to infer between-group transmission patterns, as highlighted by the presence of large well-mixed polytomies in the phylogeny. Analysing sequences from a faster mutating pathogen might enable characterization of such a between-group transmission process: although population mixing occurs rapidly, genome sequences are sufficiently divergent to capture between-group transmission patterns (high between-group transmission/high mutation rate). Though insufficient to characterize rapid mixing processes, sequences from slow-mutating pathogens still have the potential to decipher slow between-group transmission processes (low between-group transmission/low mutation rate).

Here, we study how the interaction between sampling, transmission and evolutionary processes impacts our ability to characterize transmission between population groups from sequence datasets, focusing on estimating between-group transmission rates. We focus on sequence data analysis at the consensus level. Such datasets can be analysed through phylogeny-based methods. However, establishing how these factors affect the power of tree-based methods (e.g. by influencing coalescent patterns) is delicate. Recent work has illustrated that genetic distance-based approaches can characterize transmission at the population level [2]. Such approaches provide a more straightforward angle to characterize how these many factors influence the information contained in sequence datasets. Here, we develop a conceptual framework describing the ability of characterizing pathogen spread between population groups from the analysis of pairs of genetically proximal sequences. We apply this framework to a range of pathogens, characterized by distinct evolutionary characteristics and natural history parameters, and mixing processes (between age groups and various geographic scales). From this, we identify fundamental limits in the ability of pathogen genome sequencing to capture transmission dynamics at the group level. Finally, we conduct a simulation study to characterize how sampling additionally impacts the signal contained in clusters of proximal sequences.

## 2. Methods

### (a) Problem framing

We quantify the extent to which pathogen genome sequencing is informative about between-group transmission processes. We define genomic *linkage criteria* between groups and evaluate how well they capture (true) transmission links. Specifically, we assume that two groups are genomically linked through an observed sequence pair if the genetic distance between the elements of this sequence pair lies below a defined threshold Δ. We want to determine what is the sensitivity $\eta_{\Delta }$, specificity $\chi_{\Delta }$, positive predictive value (PPV) $\phi_{\Delta }$ and accuracy $A_{\Delta }$ of this group linkage criterion.

### (b) Probabilistic framework

#### (i) Notation

Let J denote the number of between-group transmission events separating two infected individuals. We assume that they occur under a Poisson process of rate λ, where λ is the between-group transmission rate. In phylogeographic studies focusing on geographical units, λ is often referred to as the migration rate. By modelling transmission in a well-mixed population, where between-group transmission events occur independently and at a constant rate, we do not account for potential network structure (which can be important in outbreak settings such as households, schools or workplaces). This assumption should be most relevant when studying population-level transmission, where the within-group transmission probability can be treated as constant along a transmission chain and is in line with standard tree-based approaches (discrete trait analysis, structured coalescent and birth–death models), which also do not account for any network structure.

Let M denote the number of mutations between the infecting pathogens of these two infected individuals. We assume that mutation events occur under a Poisson process of rate μ, where μ is the per genome mutation rate. Let G be a random variable denoting the number of generations separating these two individuals, which we define as the number of transmission events separating these individuals along a transmission chain. We assume that the generation time follows a gamma distribution of shape α and scale β.

#### (ii) Distribution of the number of mutations and the the number of between-group transmission events conditional on the number of generations

Under these assumptions, the number of mutations M conditional on the number of generations G=g follows a negative binomial distribution of shape $r_{J|g}=\alpha g$ and probability $p_{J|g}=\frac{\beta }{\beta +\lambda }$ parameters [2]. Similarly, the number of between-group transmission events J, conditional on the number of generations G=g follows a negative binomial distribution with parameters:

$$r_{M|g}=\alpha g\;p_{M|g}=\frac{\beta }{\beta +\mu }$$

#### (iii) Distribution of the number of between-group transmission events conditional on the number of mutations

In practice, we do not observe the number of generations separating two infected individuals and are instead interested in the distribution of the number of between-group transmission events conditional on the number of mutations. Let h(k;r,p) denote the probability mass function evaluated in k of a negative binomial distribution of parameters r and p. We introduce $\pi_{g}$, the probability that two sequenced individuals are g generations apart. By integrating over the possible number of generations separating two infections, we show that (full derivation in the electronic supplementary material):

$$P[J=j|M=m]=\frac{\sum_{g\geq 1}h\left(j;\alpha g,\frac{\beta }{\beta +\lambda }\right)\cdot h\left(m;\alpha g,\frac{\beta }{\beta +\mu }\right)\cdot \pi_{g}}{\sum_{g\geq 1}h\left(m;\alpha g,\frac{\beta }{\beta +\mu }\right)\cdot \pi_{g}}$$

We assume each infection can only be sampled once, which means we do not have any pair of samples in our dataset corresponding to g=0 (0 transmission generation).

The distribution $\pi_{g}$ of the number of generations between infected individuals is impacted by several factors, including the epidemic dynamics and the sampling scheme [8]. Wohl *et al*. used a simulation-based approach to approximate this probability distribution across a range of epidemiological scenarios, characterized by their reproduction number [8]. Their empirical estimates were obtained by simulating a branching process for d=ln(1000)/ln(R) generations, (where R is the reproduction number). This corresponds to the number of generations required to reach an expected epidemic size of 1000. From this, they derive the empirical distribution of the number of generations separating two infections (which we denote ${\hat{\pi }}_{g}$). By design, the maximum number of generations separating two infected individuals in their simulations is therefore $g_{\text{max}}=2d$, which depends on the reproduction number. We fully approximate the probability P[J=j∣M=m] by replacing $\left(\pi_{g}\right)$ by the empirical probability distribution $\left({\hat{\pi }}_{g}\right)$. The probability of two infected individuals being separated by between-group transmission events and mutations thus follows:

$$P\left[J=j\right]=\sum_{g=1}^{g_{\text{max}}}h\left(j;\alpha g,\frac{\beta }{\beta +\lambda }\right)\cdot {\hat{\pi }}_{g}\;P[M=m]=\sum_{g=1}^{g_{\text{max}}}h\left(m;\alpha g,\frac{\beta }{\beta +\mu }\right)\cdot {\hat{\pi }}_{g}$$

### (c) Confusion matrix approach

#### (i) Definition

**Figure 2.**
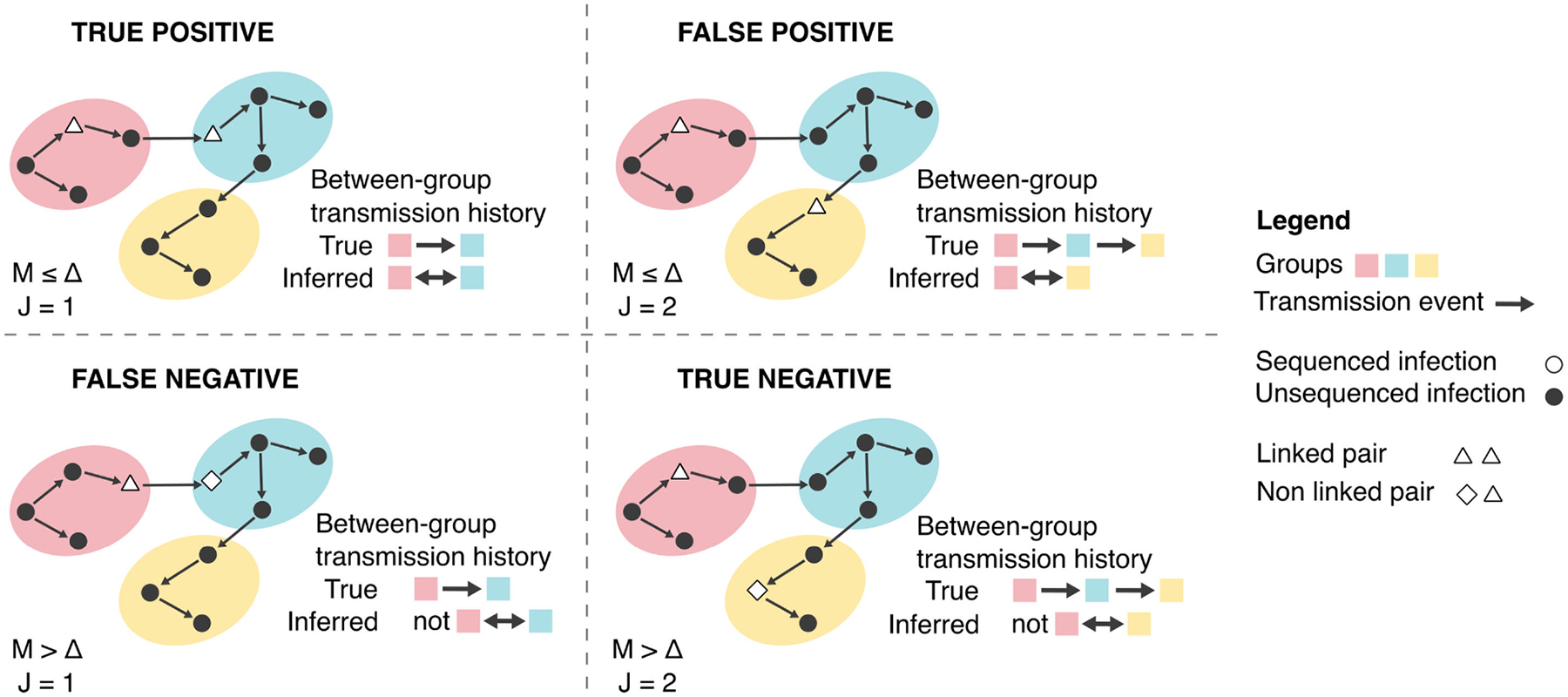
Illustration of the confusion matrix used to describe the ability of a genetic linkage criterion to capture the pathogen’s between-group transmission history. These schematics illustrate a transmission chain propagating across three different population groups, each depicted by a coloured oval shape. Group membership is based on host characteristics or sequence metadata (such as age or geographic region). Each point (circle, triangle or diamond) corresponds to an infected individual, with white filled points indicating sequenced infections and black filled points indicating infections that are not sequenced. Each diagram illustrates the example of a pair of sequences (white filled points) corresponding to a true positive, false positive, false negative or true negative. For each of these diagrams, we indicate the corresponding ‘true’ between-group transmission history between the two sequenced individuals and the history inferred from the genomic linkage criterion.

To quantify the ability of a genetic linkage criterion to characterize transmission between population groups, we use a confusion matrix approach. We classify sequence pairs depending on their ability to accurately capture between-group transmission history from their genetic sequences. Figure 2 illustrates how we define the true between-group transmission history between two sequenced individuals and the inferred between-group transmission history from sequence data. For example, on the top left, the sequences of our two sampled infections define a linked pair (the number of mutations separating their genomes is below a predefined threshold). The true transmission history between these samples is *red* → *blue*. From our linked pairs, we infer that transmission occurred between these two groups (*red* ↔ *blue*). The inferred transmission history, therefore, accurately captures the true underlying history, corresponding to a true positive (TP). A false positive (FP) corresponds to a mismatch between the inferred between-group transmission history and the actual one. Likewise, if another between-group transmission event occurred between the pair members and the pair is not linked, that is a true negative (TN), and if no other transmission event to another group occurred between the two sampled individuals and the pair is not linked, that is a false negative (FN). Our probabilistic framework enables us to define the confusion matrix coefficients (table 1). By definition, this linkage criterion does not account for transmission direction, and we focus on whether a sequence pair accurately represents the underlying between-group transmission history, regardless of directionality. Some pairs characterized by J>1 may coincide with one segment of the full between-group transmission path (e.g. transmission *red* → *blue* → *red* → *blue* between two sequences, so that *red* ↔ *blue* captures a subset of the transmission history). We deliberately classify these pairs as TN or FP (like other pairs with J>1) as they do not capture the entire between-group transmission history. This formulation provides a conservative assessment of our linkage criterion’s performance.

#### (ii) Sensitivity

The sensitivity $\eta_{\Delta }$ is the true positive rate. It measures how well our linkage criterion captures sequence pairs that reflect the true between-group transmission history. From table 1, we derive it as (see electronic supplementary material):

$$\eta_{\Delta }=P[M\leq \Delta |J\leq 1]=\sum_{d=0}^{\Delta }\frac{\left(P[J=0|M=d]+P[J=1|M=d])\cdot P[M=d\right]}{P[J=0]+P[J=1]}.$$

#### (iii) Specificity

The specificity $\chi_{\Delta }$ is the true negative rate. It measures the fraction of pairs not reflecting the true between-group transmission history that are not captured by the linkage criterion Δ. From table 1, we derive it as (see electronic supplementary material)

$$\chi_{\Delta }=P[M>\Delta |J>1]=1-\sum_{d=0}^{\Delta }\frac{\left(1-P[J=0|M=d]-P[J=1|M=d])\cdot P[M=d\right]}{1-P[J=0]-P[J=1]}.$$

#### (iv) Positive predictive value

The PPV $\phi_{\Delta }$ measures the proportion of linked pairs that correctly capture the between-group transmission history:

$$\phi_{\Delta }=P[J\leq 1|M\leq \Delta ]=\frac{P[M\leq \Delta |J\leq 1]\cdot P[J\leq 1]}{P[M\leq \Delta ]}=\eta_{\Delta }\cdot \frac{P[J\leq 1]}{P[M\leq \Delta ]}.$$

#### (v) Accuracy

Similarly, we compute the overall accuracy $A_{\Delta }$ as

$$A_{\Delta }=\frac{TP+TN}{TP+TN+FP+FN}=\eta_{\Delta }\cdot P[J\leq 1]+\chi_{\Delta }\cdot (1-P[J\leq 1]).$$

### (d) Spatial and age-based transmission processes’ characteristics

We apply our confusion matrix framework to a combination of pathogens and mixing processes to understand how analysing the pathogen genome sequences of these different pathogens can provide insights into these population processes. We focus on transmission processes between geographies and age groups. To characterize these mixing processes, we use empirical data to estimate the probability ω that a between-group transmission event occurs before a mutation event using mobility and social contact data. This enables us to estimate the probability of a movement occurring within different geographies in the USA (electronic supplementary material, table S1) and for a contact occurring within different age groups (electronic supplementary material, figures S1,S2) in Washington State. We detail our approach and the data used for this assessment in the electronic supplementary material.

#### (i) Relationship between the probability for the infectee to be in the same subgroup as the infector and the between-group transmission event rate λ

We relate the probability ω that a transmission event occurs within the same population subgroup to the between-group transmission event rate λ using

$$\omega =P[J=0|G=1]={\left(\frac{\beta }{\beta +\lambda }\right)}^{\alpha }.$$

Therefore, for a known value of ω, the corresponding between-group transmission rate is equal to:

$$\lambda =\beta \left(\omega^{-\frac{1}{\alpha }}-1\right).$$

We use ω values estimated from mobility and social contact data (see electronic supplementary material). ω describes the within-group transmission probability per transmission event. For brevity, we refer to it as ‘within-group transmission probability’ in this article.

### (e) Case study across a range of pathogens

#### (i) Evolutionary and transmission characteristics

We apply our confusion matrix approach to Ebola virus, seasonal influenza virus A/H1N1pdm and A/H3N2, measles virus, MERS-CoV, mpox virus, mumps virus, RSV-A, SARS-CoV, SARS-CoV-2 (both pre- and post-Omicron) and Zika virus. We assume that sequencing provides whole-genome sequences for all these pathogens. We use previously estimated values of the probability p that a transmission event occurs before a mutation event for these pathogens [9]. We explore an additional scenario wherein only the HA segment of the influenza A/H3N2 virus is analysed (corresponding to p of 0.92). The shorter genomic target in HA represents a scenario with a reduced per-genome mutation probability.

#### (ii) Epidemiological scenarios

We show above that sensitivity, specificity and PPV depend on the distribution of the number of generations separating two individuals picked at random in the population. We use the empirical distributions generated by Wohl *et al*. [8] for reproduction numbers R of 1.3, 1.5 and 1.8, with results for R of 1.3 described in the main text and for R of 1.5 and 1.8 presented in electronic supplementary material, figures S3,S4.

### (f) Characterizing the parameter space across pathogens

To comprehensively explore trends across pathogens and between-group transmission processes, we apply our confusion matrix approach to a range of evolutionary and mixing parameters. We consider a pathogen with a generation time of mean 4.9 days and standard deviation of 4.8 days. This corresponds to the values we used for SARS-CoV-2 (Omicron variant). Assuming a gamma-distributed generation time, this corresponds to a shape of 1.04 and a scale of 0.21 days. This parametrization is arbitrary and simply provides a direct way to map evolutionary rates μ to values of the probability that transmission occurs before mutation p and mixing rates λ to values of within-group transmission probability ω. We then compute sensitivity, specificity and PPV by varying p and ω between 0.01 and 0.99 with an increment of 0.01.

### (g) Simulation study to explore the relationship between power and sample size

We evaluate how sample size influences the ability to characterize transmission patterns between groups from sequence data by performing a simulation study using the ReMASTER BEAST2 package [10] (full details in electronic supplementary material). We simulate pathogen sequencing over the course of an outbreak and evaluate how the sampling intensity (measured by the sequencing fraction $p_{\text{seq}}$), the between-group transmission process (measured by the within-group transmission probability ω) and the evolutionary process (measured by the probability p that transmission occurs before mutation) impact our ability to draw inference from genetically proximal sequences. To assess the ability of sequences below a given genetic distance threshold Δ to capture mixing patterns, we compute a relative risk (RR) metric that was introduced in prior work and shown to capture SARS-CoV-2 transmission patterns between age groups and geographies [2]. For each combination of $p_{\text{seq}},\omega$ and p, we simulate 50 outbreaks with associated sequence data and compute relative risks for thresholds ranging between 0 and 15. We then compute the median Spearman correlation coefficient between RRs and daily between-group transmission probabilities across the 50 replicate simulations. To characterize the best inference performance for a given sequencing effort, we compute the maximum median correlation across Δ ranging between 0 and 15 as

$$\rho^{50,\;\text{max}}\left(p_{\text{seq}},\;\omega ,\;p\right)=\underset{0\leq \Delta \leq 15}{\text{max}}\;\rho^{50}\left(p_{\text{seq}},\;\omega ,\;p,\;\Delta \right).$$

We then characterize the sequencing effort required to reach a correlation threshold τ (50 and 90%) for each (ω,p) combination as

$$p_{\text{seq}}^{\text{required}\;\tau }(\omega ,\;p)=\underset{p_{\text{seq}}\in \{0.001,\;0.005,\;0.01,\;0.05\}}{\text{min}}\left\{p_{\text{seq}}|\rho^{50,\;\text{max}}\left(p_{\text{seq}},\;\omega ,\;p\right)\geq \tau \right\}.$$

## 3. Results

### (a) Rescaling of evolutionary and mixing rates by generation times

As we rely on genetic distances between consensus sequences, the signal for between-group transmission comes from the occurrence (or lack of occurrence) of mutations between pairs of infected individuals. This signal is determined both by the rate at which mutations accumulate in pathogen genomes and the typical delay between successive infections, which defines a window of opportunity for mutations to occur. Because the generation time varies widely between pathogens, the genome-wide mutation rate μ thus does not directly map to the expected divergence between transmission pairs (electronic supplementary material, figure S5A). To account for this, we present our results as a function of the probability p that transmission occurs before mutation, which allows us to rescale the mutation rate by the generation time distribution and directly captures the expected genetic divergence between transmission pairs (electronic supplementary material, figure S5B). Electronic supplementary material, figure S5C, illustrates how the relationship between p and μ is modulated by the generation time distribution. We use the same scaling approach to characterize mixing scales by relying on the within-group transmission probability ω, which corresponds to the probability that a transmission event occurs before a between-group transmission event. Electronic supplementary material, figure S5D, depicts the relationship between ω and the between-group transmission rate λ.

### (b) Factors impacting the ability of a genetic linkage criterion of capturing transmission between population groups

**Figure 3.**
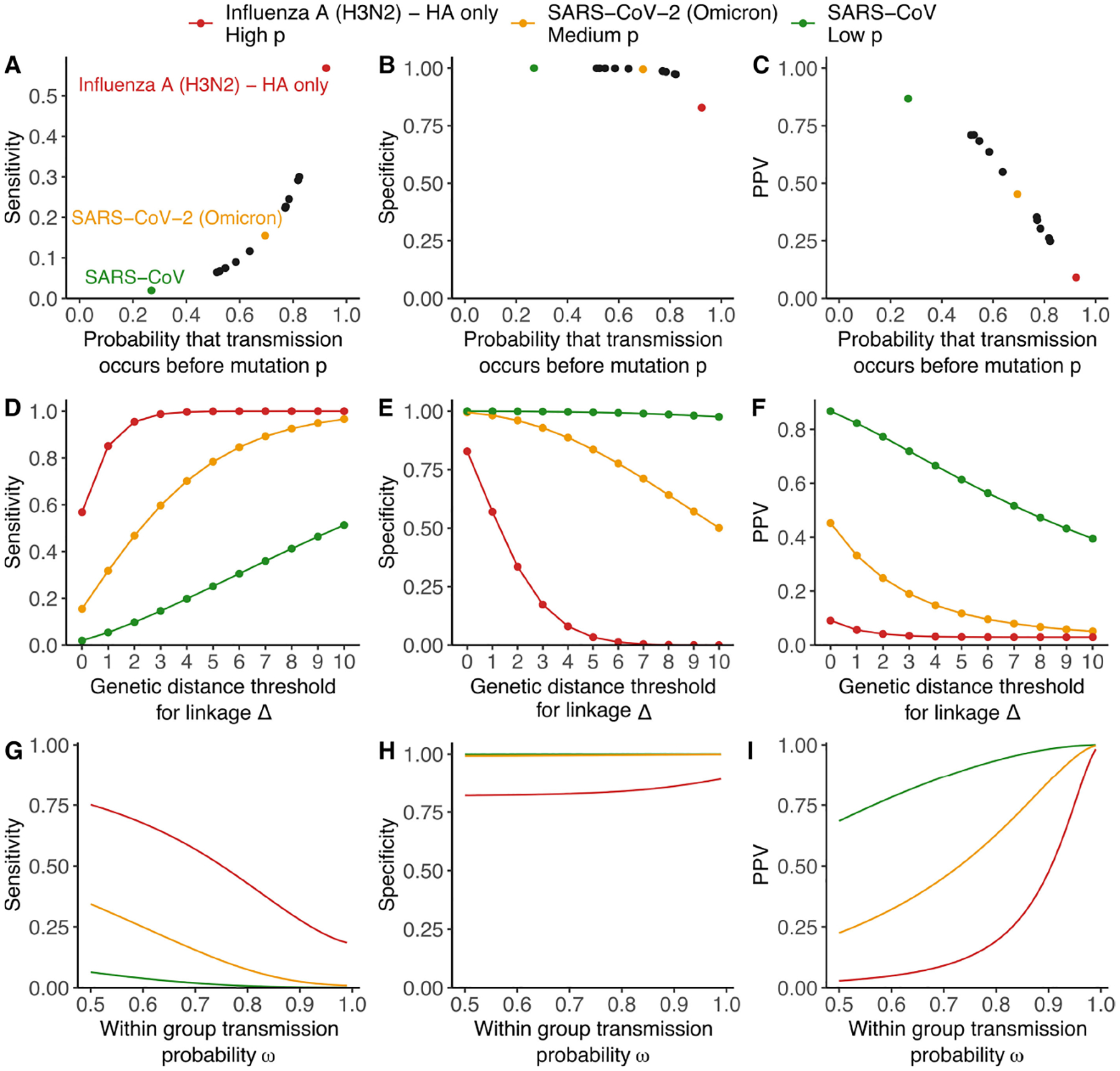
Impact of the mutation and mixing processes and the genetic distance threshold on the sensitivity, specificity and PPV. (A) Sensitivity, (B) specificity and (C) PPV of a linkage criterion defined by Δ=0 assuming a within-group transmission probability ω of 0.7 across pathogens and depicted as a function of the probability that a transmission event occurs before a mutation event across pathogens. (D) Sensitivity, (E) specificity and (F) PPV as a function of the distance threshold used to define the linkage criterion and assuming a within-group transmission probability ω of 0.7. (G) Sensitivity, (H) specificity and (I) PPV of a linkage criterion defined by Δ=0 as a function of the within-group transmission probability ω.

We find that the linkage criterion’s performance varies across pathogens and is determined by the relative time scale at which mutation and transmission events occur (figure 3A–C). For example, the sensitivity increases as the probability p that transmission occurs before mutation increases (corresponding to slower mutating pathogens, when scaling the mutation rate with the time it takes for each transmission generation to occur), while the specificity and the PPV decrease with p.

**Figure 4.**
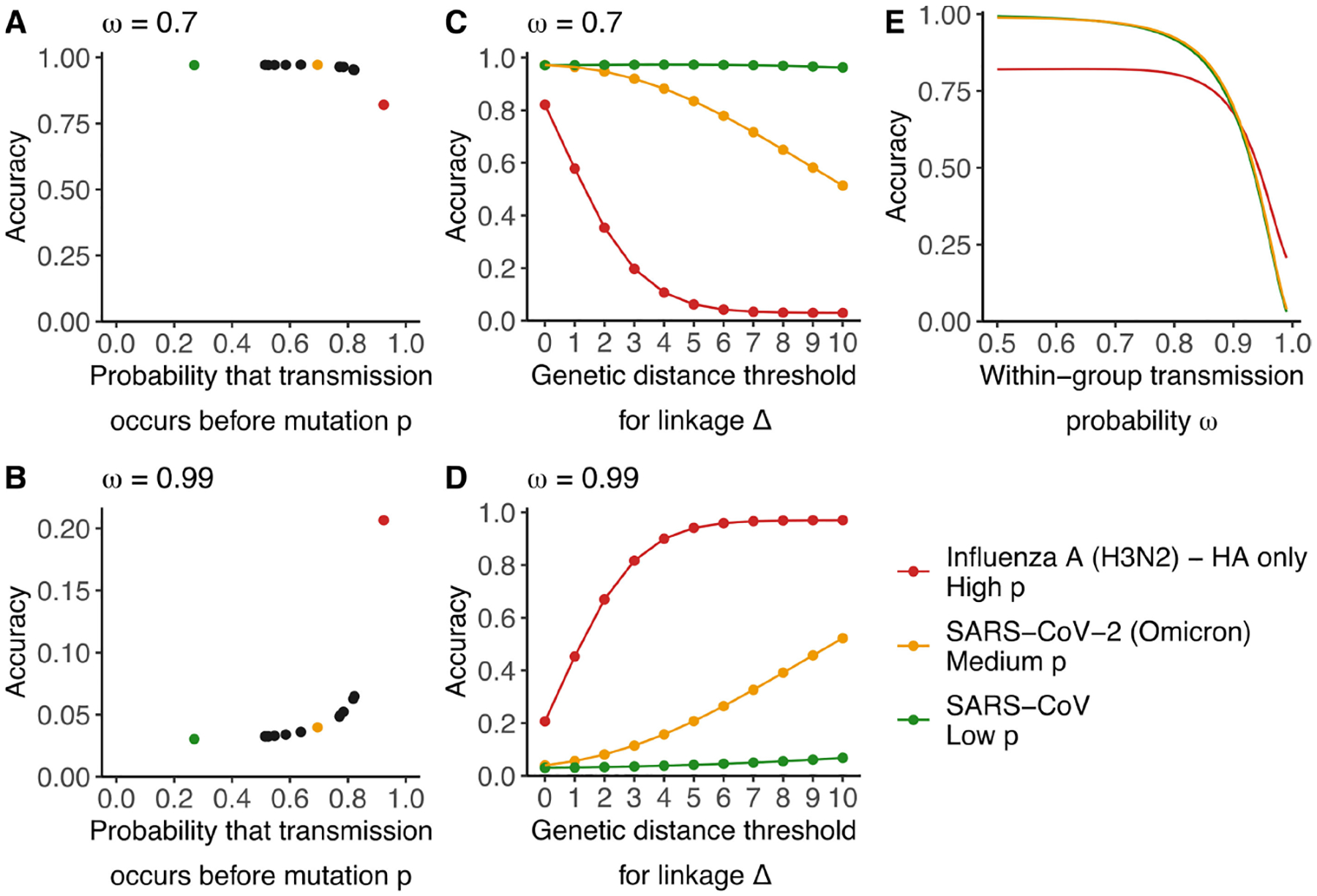
Impact of the mutation and mixing processes, and the genetic distance threshold on the linkage criterion’s accuracy. Impact of the probability that transmission occurs before mutation on the accuracy of a linkage criterion defined by Δ=0 assuming a within-group transmission probability ω of (A) 0.7 and (B) 0.99. Impact of the distance threshold Δ on the linkage criterion’s accuracy assuming a within-group transmission probability ω of (C) 0.7 and (D) 0.99. (E) Impact of the within-group transmission probability ω on the accuracy of a linkage criterion defined by Δ=0.

To further explore how other parameters impact linkage performance, we focus on a subset of the pathogens depicted in figure 3A–C. We select this subset to ensure coverage of the full range of p: SARS-CoV (low p), SARS-CoV-2 (Omicron period, intermediate p) and influenza A/H3N2 (HA only, high p). Figure 4D–F depicts how varying the genetic distance threshold used to define the criterion impacts overall performance across these pathogens. Specificity and PPV are always maximized at low thresholds, while sensitivity increases as the threshold is relaxed. This is expected as a lower threshold captures infections that are more epidemiologically linked and therefore less likely to misrepresent between-group transmission history. These lower thresholds, however, come at a sensitivity cost, as some relevant pairs are not captured by a more conservative criterion.

We expect the pace at which between-group transmission events occur to impact linkage performance. In figure 3G–I, we explore the within-group transmission probability’s impact on linkage performance. Faster mixing processes (characterized by lower within-group transmission probability values) are associated with higher sensitivities and lower specificities and PPVs than slower mixing processes, for a specified threshold Δ used to define linkage. This is expected because the probability for a pathogen to have moved several times between groups increases as the within-group transmission probability decreases (faster mixing processes), thereby leading to capturing pairs that are less representative of the between-group transmission history.

We also compute the linkage criterion’s overall accuracy. For lower values of the within-group transmission probability ω, the accuracy increases with the probability that transmission occurs before mutation and decreases with the distance threshold Δ, following similar trends as the linkage’s specificity (figure 4). For higher values of ω, corresponding to slower mixing processes, the accuracy follows inverse trends, behaving more similarly to the linkage’s sensitivity. This is expected as the accuracy is computed as a combination of the sensitivity and the specificity, with weights attributed to each metric related to the probability that a pair of sequences is separated by less than one between-group transmission event (see Methods), which depends on ω. Unlike PPV, which measures the informativeness of linked pairs, accuracy aggregates performance over both linked and unlinked pairs and therefore depends on the frequency of between-group transmission events.

Linkage performance is impacted by assumptions regarding the distribution of the number of generations separating two infected individuals in the population, and therefore the reproduction number. However, a sensitivity analysis varying the reproduction number shows that overall trends are maintained (electronic supplementary material, figure S4).

Overall, these findings demonstrate that the ability for pathogen genome sequence data to characterize transmission between population groups from genetically proximal sequences depends on the interplay between evolutionary, transmission and mixing processes.

### (c) Limits of genetic sequence data in their ability to characterize population processes

**Figure 5.**
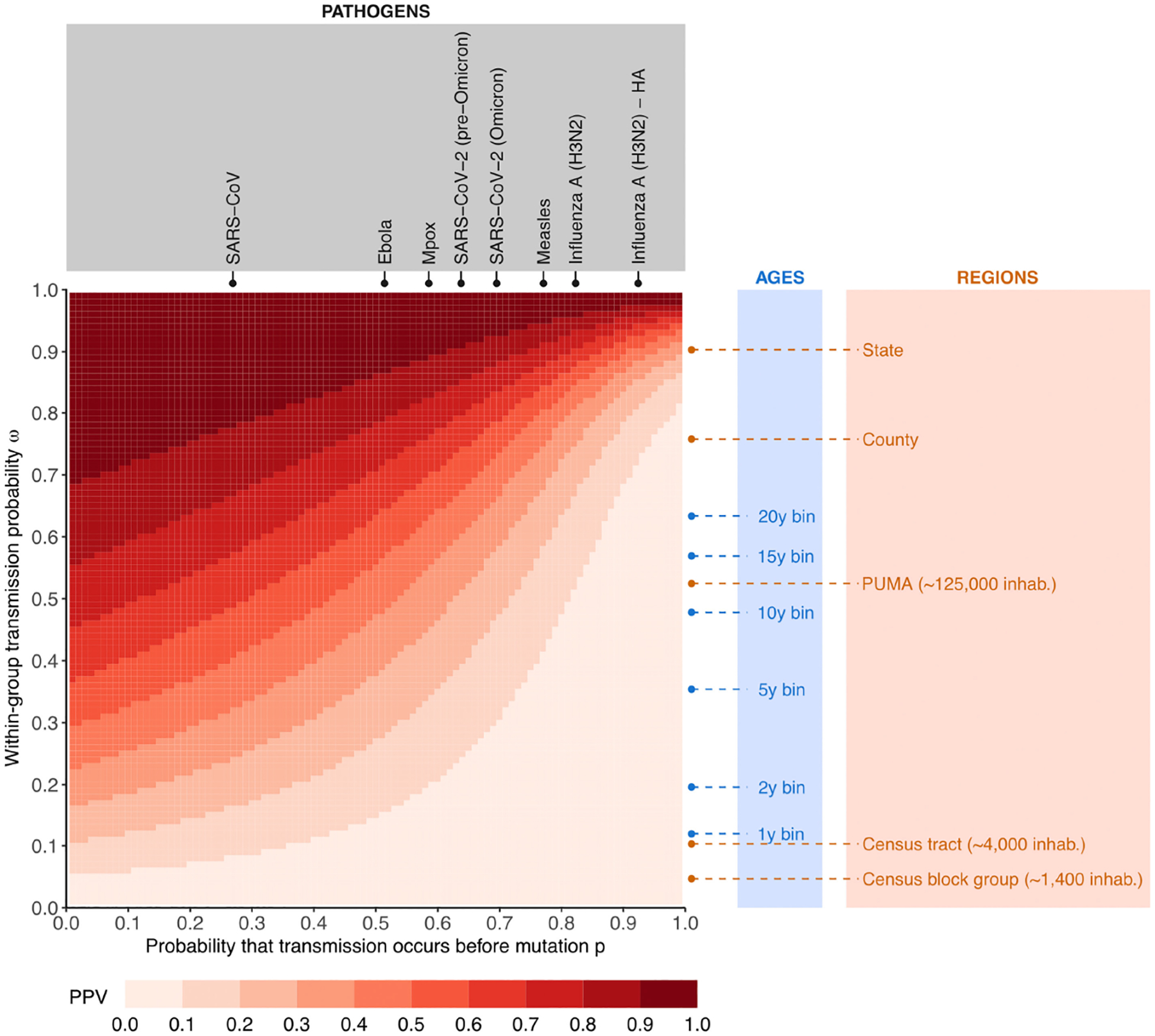
The pace at which mutation and between-group transmission events occur determines the extent to which pathogen sequences are informative about mixing processes. The heatmap depicts the PPV associated with a threshold Δ of 0 as a function of the probability that a transmission event occurs before a mutation event p and the within-group transmission probability ω. For context, we indicate on the top values for p across a range of pathogens and on the right values for ω across a range of mixing processes. Blue indicates within-age group transmission probability ω for age groups defined by a range of binning widths (1 year binning to 20 year binning) using WA social contact data. Orange indicates within-region transmission probabilities estimated using mobile phone movement data for US regions of varying sizes: states, counties, Public Use Microdata Areas (PUMAs), census tracts and census block groups (CBGs). Details about how we estimate such values are available in the electronic supplementary material. Values are computed considering a pathogen with a mean generation time of 4.9 days and a standard deviation of 4.8 days and a reproduction number of 1.3 (baseline epidemiological scenario).

The PPV describes how often a pair of sequences separated by a Hamming distance less than Δ accurately captures between-group transmission history. For a given pathogen (characterized by its probability p that transmission occurs before mutation) and transmission process (characterized by its probability ω that transmission occurs within the same group), this PPV is highest for a distance threshold Δ of 0 (figure 3F). To explore the ability of consensus genome sequences in characterizing population processes, we computed the PPV for a threshold Δ of 0 as a function of both p and ω (figure 5). To facilitate interpretability, we indicate on the left of the figure how different mixing processes map to within-group transmission probabilities (ω) and on the top how different pathogens map to values of p.

The PPV for a genetic distance threshold Δ of 0 varies considerably across the parameter space. In our baseline epidemiological scenario, for a pathogen characterized by a p of 0.2, we estimate a PPV of 0.28 for a fast-mixing transmission process (ω=0.2) and a PPV of 0.93 for a slower mixing process (ω=0.8). By contrast, these PPVs drop to 0.02 (ω=0.2) and 0.44 (ω=0.8) for a pathogen characterized by a p of 0.8. We identify a region of low PPV in the phylogeographic parameter space, primarily in the region wherein values of p are higher than values of ω (lighter red colours in figure 5). This corresponds to combinations of pathogens and mixing processes for which analysing consensus sequence data does not provide sufficient resolution to characterize the corresponding mixing process. Each pathogen is therefore associated with a horizon of observability regarding population mixing processes that depends on the pace at which mutations accumulate within its genome.

Classifying pairs of sequences separated by a genetic distance below Δ(M≤Δ) but between which no between-group transmission event occurred (J=0) rather as TNs than TPs (to focus on between-group transmission events) leads to similar conclusions (electronic supplementary material, figure S6).

### (d) Trade-off between sample size and positive predictive value

**Figure 6.**
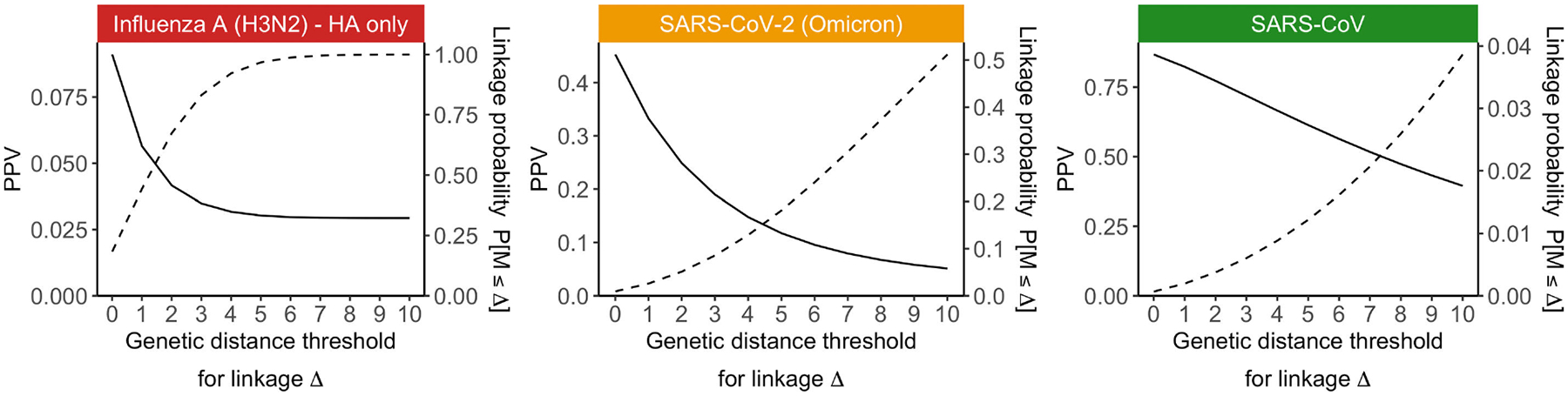
Sample size impacts the distance threshold Δ that maximizes study power. Impact of the genetic distance threshold Δ on the PPV (solid line) and the linkage probability P[M≤Δ] (dashed line) assuming a within-group transmission probability ω of 0.7 across pathogens.

A high PPV ensures the signal from linked sequence pairs is as specific as possible and captures the true between-group transmission history. This PPV is maximized at low distance thresholds Δ, but this comes at a cost of reducing the number of pairs used in the analysis, as lower thresholds result in decreasing linkage probability (figure 6). This underlines that the performance of our group linkage criterion cannot be considered in isolation from the composition and size of the dataset being studied. A low linkage probability and a high PPV may be preferred in a large dataset but may not be useful when analysing a smaller set of sequences, wherein only a few sequence pairs ultimately meet the linkage criterion. The PPV, therefore, quantifies the informativeness of genome sequences about population mixing processes in situations where the sample size is large enough for low thresholds not to yield a critically low number of linked pairs.

**Figure 7.**
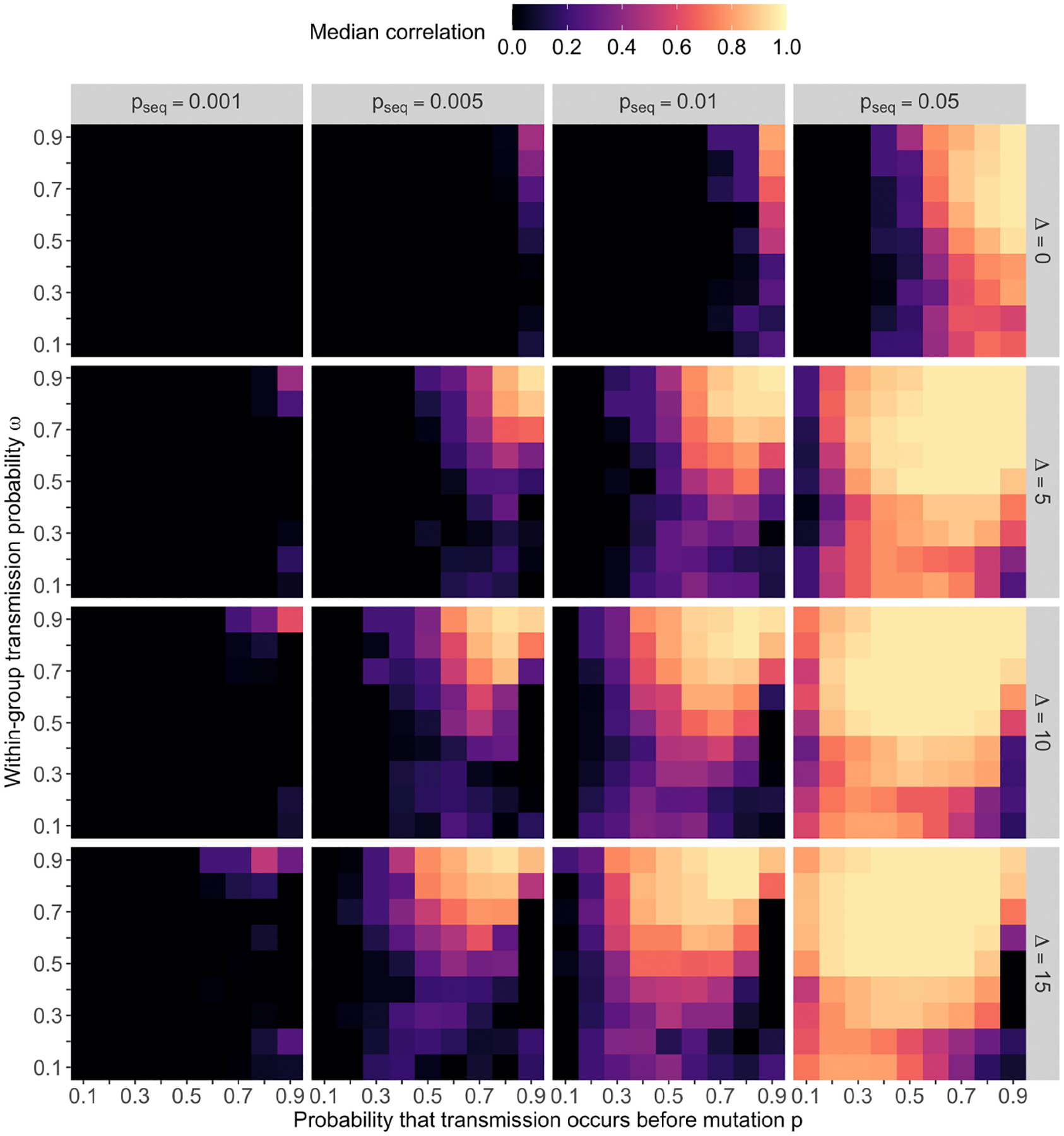
Impact of sample size and genetic distance threshold on inference accuracy. Median Spearman correlation coefficient (across 50 replicate simulations) between the RR of observing sequences less than Δ mutations away (rows) and transmission probabilities between groups, across different sequencing fractions $p_{\text{seq}}$ (columns). Results are displayed as a function of the probability p that transmission occurs before mutation and within-group transmission probability ω. When the median correlation is lower than 0, we display it as black (corresponding to 0) to improve visualization of positive median correlation values.

To investigate how sample size impacts the ability to characterize mixing processes from pairs of genetically proximal sequences, we simulate synthetic outbreaks and vary the rate at which infected individuals are sequenced. We then compute the correlation between the RR of observing sequence pairs separated by less than Δ mutations and the transmission probability between these subgroups [2]. This RR metric quantifies the extent to which pairs of sequences lying below a distance threshold Δ are enriched in sequences coming from specific subgroups. Prior work demonstrated that this metric could serve as an alternative to traditional tree-based phylogeographic methods [2], and the median correlation reported in figure 7 corresponds to a measure of method accuracy.

**Figure 8.**
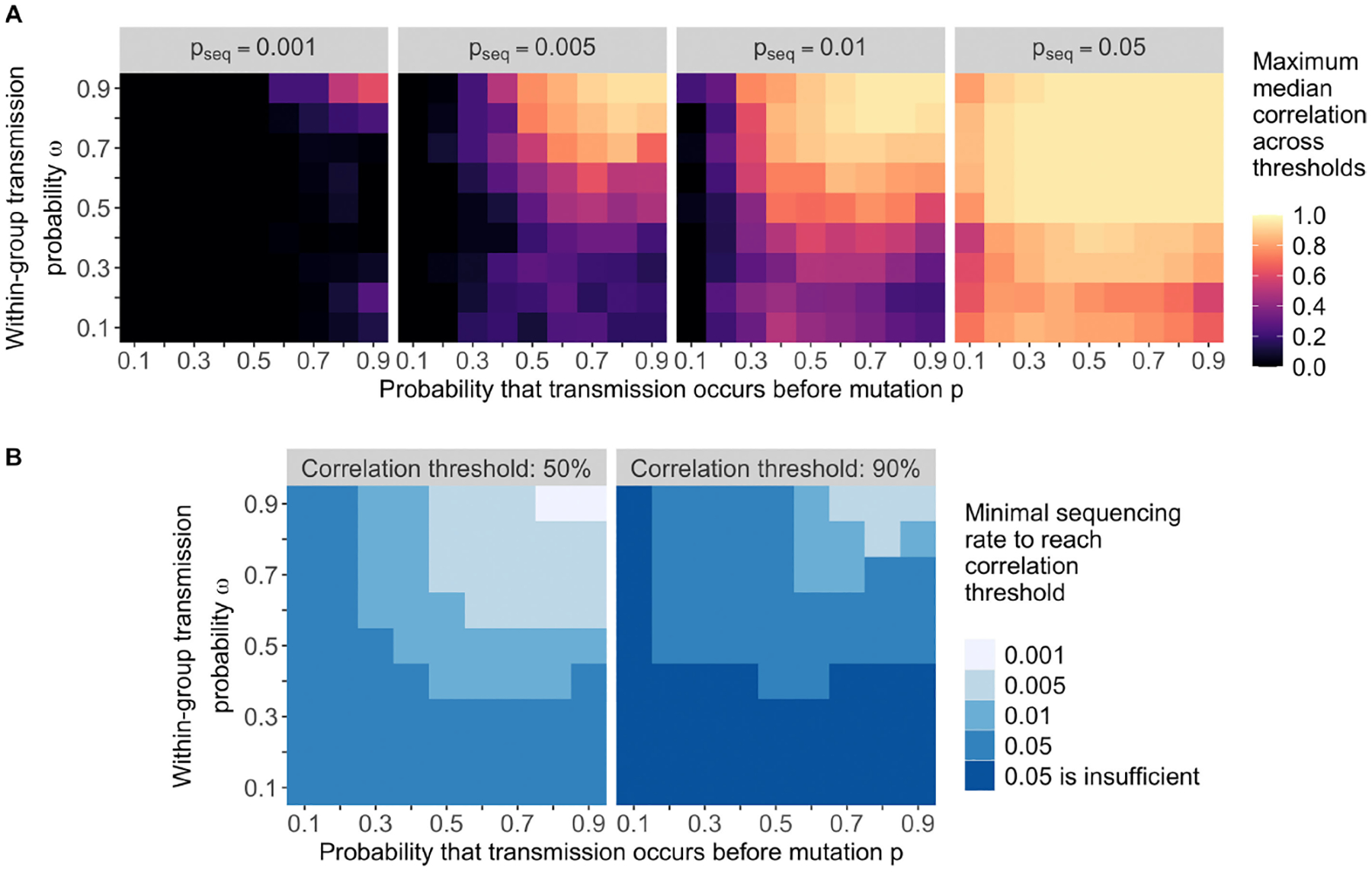
Minimal sampling effort necessary to reach a given inference accuracy. (A) Maximum median correlation between the RR of observing sequences less than Δ mutations away and between-group transmission probabilities as a function of p and ω, across different sequencing fractions $p_{\text{seq}}$. This maximum correlation is computed across distance thresholds Δ ranging between 0 and 15. (B). Minimal sequencing fraction $p_{\text{seq}}$ required to reach a maximum median correlation of 50 or 90%, as a function of p and ω.

Sample size is another important factor influencing study power, with low sequencing fractions being associated with a lower accuracy (figure 7). Despite the PPV being highest for a genetic distance threshold Δ=0, relying on this threshold is not sufficient to characterize mixing processes at low sequencing rates (top left facet in figure 7). Considering less restrictive distance thresholds can increase study power (bottom left facet in figure 7), by increasing the number of sequence pairs analysed (electronic supplementary material, figure S7). However, the number of sequences available and analysed imposes an upper bound on inference accuracy, regardless of the distance thresholds (figure 8A). Figure 5 highlights a fundamental limit for characterizing between-group transmission processes, determined by the relative pace at which mutation and between-group transmission events occurred. Here, we show that study design imposes an additional constraint. While it is theoretically possible to characterize a transmission process characterized by a within-group transmission probability ω~0.5 from a pathogen characterized by p~0.7 (which is similar to assessing SARS-CoV-2 transmission between age groups defined in decade increments; figure 5), our simulations highlight that this requires a sufficiently high level of sequencing. For example, in our four-group transmission simulations, sequencing 1% of the infected population would not yield an inference accuracy greater than 90%, and one would need to rely on at least 5% of infections being sequenced to draw such inferences (figure 8B).

Overall, the ability to decipher between-group transmission from proximal sequences is influenced by the interaction between sampling intensity and the relative time scale at which mutations and between-group transmission events occur.

## 4. Discussion

Here, we explore how the complex interplay between transmission, population mixing, mutation and sampling impacts the informativeness of sequence datasets about population-level epidemic processes from proximal sequence pairs. First, we use a confusion matrix approach to quantify whether sequence pairs are consistent with the underlying between-group transmission history. This succinct formulation enables us to comprehensively explore the space of possible phylogeographic analyses by explicitly incorporating the pace at which mutations accumulate within pathogen genomes (measured by the probability p that transmission occurs before mutation) and the pace at which pathogens move between groups (measured by the within-group transmission probability ω). Our analyses reveal an inherent limit to the resolution genomic data can provide, particularly when between-group transmission events occur faster than the accumulation of mutations within pathogen genomes. Second, we complement this theoretical framework with simulations to investigate how sampling effort and study design impact the accuracy and power of studies based on genetically proximal sequences. These simulations reveal a second fundamental constraint, with sparser sampling decreasing the ability of pathogen sequences to decipher transmission between groups.

Our confusion matrix formulation relies on a few assumptions. First, we parametrized the transmission process with a single parameter (the within-group transmission probability ω), which we estimate for a few transmission processes (electronic supplementary material, table S1, figure S2). In practice, the within-group transmission probability varies across groups (electronic supplementary material, figure S1), socio-demographic settings [11,12] or with changing immunity profiles. Second, we use the probability that transmission occurs before mutation to describe the probability for an infector and an infectee to have the same consensus sequence. This is a valid assumption for pathogens causing acute infections characterized by narrow transmission bottlenecks [9]. Pairs of consensus sequences from pathogens causing chronic infections also contain valuable information about epidemiological processes [13,14]. Determining how the trade-offs we identified translate to such pathogens would be interesting. Finally, we explore how sampling impacts the informativeness of pathogen genome datasets by simulating the spread and sequencing of a pathogen between four groups of a population. While we expect the patterns we describe in figures 7 and 8 to hold for other processes, we could not derive a formula for the sequencing fraction or sample size required as a function of simply p and ω. Such a quantity would be impacted by factors that are not fully captured by our simple parametrization (including the number of groups, size of groups and between-group transmission rates).

We focus on the information contained by pairs of proximal consensus sequences to infer a between-group transmission matrix. We thus do not capture richer information contained by genomic data (e.g. derived sequences, tree branching patterns and sampling dates). Moreover, pathogen genomes can provide information about other targets, such as introduction patterns, transmission direction, effective population sizes or emergence time, which we do not explore here. However, we expect the key constraints and trends we identified to be relevant for methods leveraging genomic data differently: if between-group transmission events occur much faster than mutations, genomes will contain little information to characterize the between-group transmission process. This provides a necessary (though not sufficient) condition for the inference of between-group transmission rates from sequence data. Overall, while more sophisticated ways of leveraging genomic data may refine inference about transmission processes, some inherent limitations will persist. Further work directly quantifying such trade-offs for other phylogeographic approaches would be particularly interesting, for example, by assessing how consensus sequence pairs’ analysis could further enable characterizing transmission direction. Awareness of such constraints during study design and analysis is critical to avoid false confidence in the resulting inferences. This work emphasizes that the time scale at which between-group transmission occurs imposes an upper bound on the time scale at which genetic variation should be observed to be informative and complements existing literature on the ability to characterize epidemiological processes [3,5–7,15].

By summarizing between-group transmission with a single parameter (the within-group transmission probability ω), we do not capture the full structure of group mixing. For example, increasing the number of groups at fixed ω typically decreases between-group transmission probabilities and increases sample size requirements to fully characterize between-group transmission. Study-specific simulation exercises (similar to the one reported in figures 7 and 8) can help define expectations for how binning choices, distance thresholds Δ and sequencing density impact one’s ability to draw inferences. While future work should aim at providing robust guidance on power and sample size requirements to characterize population transmission processes, our conceptual framework provides intuition and identifies actionable levers for modulating the power of genomic epidemiological studies. Sample size and sequencing density are major determinants of the power of pathogen genomic studies [8,16] aimed at quantifying between-group transmission from proximal sequences (figures 7 and 8). However, genomic datasets used to perform such analyses are often repurposed from surveillance efforts or studies not initially aimed at quantifying transmission between groups. Increasing sample size to a desirable level might thus not be feasible, particularly for retrospective studies. One alternative is to modify key parameters’ values (within-group transmission probability ω and probability that transmission occurs before mutation p) through study design choices. For example, aggregating individuals into broader population groups both increases ω (figure 5) and decreases the number of mixing rates to estimate. Using WA contact data, we find that analysing age groups in 10 year age bins instead of 5 year ones increases the within-group transmission probability ω from 0.35 to 0.48 (figure 5; electronic supplementary material, figure S2). Considering spatial spread, aggregating individuals at the PUMA level (around 125 000 inhabitants per PUMA) instead of at the census block group level (around 1400 inhabitants) increases ω from 0.05 to 0.52 (figure 5; electronic supplementary material, table S1). The temporal resolution contained in pathogen sequences is also impacted by the analysed genome’s length, as emphasized by prior work characterizing the value of whole-genome trees relative to gene-specific trees in resolving outbreaks in space and time [7]. In our framework, this would be similar to considering a pathogen characterized by a lower p. For example, influenza A/H3N2 is characterized by a p of 0.82 when concatenating all segments [9] whereas p increases to 0.92 when analysing HA segments only. This is congruent with one mutation occurring, on average, every 19 days across the whole genome versus every 48 days for HA segments only.

The inherent limit we identified in analysing consensus sequences to characterize transmission between population groups underscores the value of developing methods leveraging identical or nearly identical sequences, particularly in settings characterized by rapid between-group mixing. Such methods can enable us to get as close as possible to that limit, and prior work has shown promising results to characterize spatial and social mixing from identical SARS-CoV-2 pairs [2]. However, we showed that even identical sequences may carry insufficient information to reliably estimate population transmission patterns (figure 5), particularly when mixing occurs rapidly with respect to mutations. Approaches explicitly leveraging within-host diversity and deep sequencing (thus capturing faster occurring evolutionary events) could effectively decrease the value of p and have the potential to overcome this limitation.

Overall, our work reveals inherent *horizons of observability* associated with the inference of between-group transmission processes from genomic data that depend on the complex interplay between study design and the relative time scale at which mutation and between-group transmission events occur.

## Supplementary Material

Supplement

Electronic supplementary material is available online at https://doi.org/10.6084/m9.figshare.c.8330939.

## Figures and Tables

**Table 1.** Confusion matrix coefficient for a linkage criterion based on a genetic distance threshold 𝚫.

	J≤1	J>1
M≤Δ	true positive (TP)	false positive (FP)
M>Δ	false negative (FN)	true negative (TN)

## Data Availability

The final version of the GitHub repository has been archived on Zenodo [17]. Code to reproduce analyses is available on GitHub at https://github.com/blab/phylogeo-signal. Supplementary material is available online [18].

## References

[1] Bedford T 2015 Global circulation patterns of seasonal influenza viruses vary with antigenic drift. Nature New Biol. 523, 217–220. (doi:10.1038/nature14460) PMC4499780 26053121

[2] Tran-Kiem C 2025 Fine-scale patterns of SARS-CoV-2 spread from identical pathogen sequences. Nature 640, 176–185. (doi:10.1038/s41586-025-08637-4) 40044856 PMC11964829

[3] Grubaugh ND, Ladner JT, Lemey P, Pybus OG, Rambaut A, Holmes EC, Andersen KG. 2019 Tracking virus outbreaks in the twenty-first century. Nat. Microbiol. 4, 10–19. (doi:10.1038/s41564-018-0296-2) 30546099 PMC6345516

[4] Grenfell BT 2004 Unifying the epidemiological and evolutionary dynamics of pathogens. Science 303, 327–332. (doi:10.1126/science.1090727) 14726583

[5] Campbell F, Strang C, Ferguson N, Cori A, Jombart T. 2018 When are pathogen genome sequences informative of transmission events? PLoS Pathog. 14, e1006885. (doi:10.1371/journal.ppat.1006885) 29420641 PMC5821398

[6] Biek R 2015 Measurably evolving pathogens in the genomic era. Trends Ecol. Evol 30, 306–313. (doi:10.1016/j.tree.2015.03.009) 25887947 PMC4457702

[7] Dudas G, Bedford T. 2019 The ability of single genes vs full genomes to resolve time and space in outbreak analysis. BMC Evol. Biol 19, 232. (doi: 10.1186/s12862-019-1567-0) 31878875 PMC6933756

[8] Wohl S, Giles JR, Lessler J. 2021 Sample size calculation for phylogenetic case linkage. PLoS Comput. Biol. 17, e1009182. (doi:10.1371/journal.pcbi.1009182) 34228722 PMC8284614

[9] Tran-Kiem C, Bedford TPN. 2024 Estimating the reproduction number and transmission heterogeneity from the size distribution of clusters of identical pathogen sequences. Proc. Natl Acad. Sci. USA 121, e2305299121. (doi:10.1073/pnas.2305299121) 38568971 PMC11009662

[10] Vaughan TG. 2024 ReMASTER: improved phylodynamic simulation for BEAST 2.7. Bioinformatics 40, btae015. (doi:10.1093/bioinformatics/btae015) 38195927 PMC10796175

[11] Read JM, Lessler J, Riley S, Wang S, Tan LJ, Kwok KO, Guan Y, Jiang CQ, Cummings DAT. 2014 Social mixing patterns in rural and urban areas of southern China. Proc. R. Soc. B 281, 20140268. (doi:10.1098/rspb.2014.0268) PMC4024290 24789897

[12] Di Domenico L 2025 Individual and neighborhood based socioeconomic factors relevant for contact behaviour and epidemic control. Commun Med 6, article 26. (doi:10.1038/s43856-025-01282-y) PMC12808736 41381877

[13] Grabowski MK 2014 The role of viral introductions in sustaining community-based HIV epidemics in rural Uganda: evidence from spatial clustering, phylogenetics, and egocentric transmission models. PLoS Med. 11, e1001610. (doi:10.1371/journal.pmed.1001610) 24595023 PMC3942316

[14] Iles JC 2014 Phylogeography and epidemic history of hepatitis C virus genotype 4 in Africa. Virology (Auckl) 464–465, 233–243. (doi:10.1016/j.virol.2014.07.006) PMC4162651 25105489

[15] Chen Z, Lemey P, Yu H. 2024 Approaches and challenges to inferring the geographical source of infectious disease outbreaks using genomic data. Lancet Microbe 5, e81–e92. (doi:10.1016/S2666-5247(23)00296-3) 38042165

[16] D’Agostino McGowan L, Wohl S, Lessler J. 2024 Power and sample size calculations for testing the ratio of reproductive values in phylogenetic samples. Am. J. Epidemiol. 194, 2367–2375. (doi:10.1093/aje/kwae378) PMC12342956 39390641

[17] Tran-Kiem C. 2026 blab/phylogeo-signal: code release associated with accepted paper (v1-corr2). Zenodo (doi:10.5281/zenodo.18274651)

[18] Tran-Kiem C, Perofsky AC, Lessler J, Bedford T. 2026 Supplementary material from: Characterizing the informativeness of pathogen genome sequence datasets about transmission between population groups. Figshare. (doi:10.6084/m9.figshare.c.8330939) PMC13466627 41844245

