# Supplementary material for: Characterizing the informativeness of pathogen genome sequence datasets about transmission between population groups
Source: Proc Biol Sci. Author manuscript; Available in PMC 2026 Aug 12. (PMC13466627; doi:10.1098/rspb.2025.2983)
Supplement: Supplementary file 1 [file NIHMS2200564-supplement-Supplement.pdf]

Supplementary information for: Characterizing the informativeness of pathogen genome sequence datasets about transmission between population groups

Cécile Tran-Kiem[1], Amanda C. Perofsky[2], Justin Lessler[3,4], Trevor Bedford[1,5]

1. Vaccine and Infectious Diseases Division, Fred Hutchinson Cancer Center, Seattle, WA, USA
2. Fogarty International Center, US National Institutes of Health, Bethesda, MD, USA
3. University of North Carolina at Chapel Hill, Chapel Hill, NC, USA
4. Johns Hopkins Bloomberg School of Public Health, Baltimore, MD, USA
5. Howard Hughes Medical Institute, Seattle, WA, USA

Published in Proceedings of the Royal Society B: Biological Sciences.
doi.org/10.1098/rspb.2025.2983

Supplementary methods

All notations are defined in the main text. To facilitate navigation, a summary of these notations is available in the following table

Parameter	Description
Random variables	
J	Number of between-group transmission events separating two infections
M	Number of mutations separating two infections
G	Number of generations separating two infections
Parametrization of the random variables' distribution	
π_g	Probability for two individuals of being separated by g generations
λ	Between-group transmission event rate
μ	Mutation rate
α	Shape parameter for the Gamma distributed generation time
β	Scale parameter for the Gamma distributed generation time
p	Probability that a transmission event occurs before a mutation one.
ω	Within-group transmission probability per transmission event
Parameters associated with the confusion matrix	
Δ	Genetic distance threshold used to define the linkage criterion
η_Δ	Sensitivity of a linkage criterion defined by a Δ threshold
χ_Δ	Specificity of a linkage criterion defined by a Δ threshold
ϕ_Δ	Positive predictive value of a linkage criterion defined by a Δ threshold

Probabilistic framework detailed derivation

Distribution of the number of between-group transmission events conditional on the number of generations.

This is similar to the derivations made in Tran-Kiem et al[1] to calculate the distribution of the number of mutations conditional on the number of generations. Let T^{evo} denote the

evolutionary time separating the two individuals we are considering. As the number of between-group transmission events follows a Poisson process of rate λ, we have:

$$J \sim \mathcal{P}(\lambda T^{evo})$$

Assuming independence of successive transmission events and because we assumed that the generation time follows a Gamma distribution of parameter (α, β), the time between g successive generations follows a Gamma distribution of shape αg and scale β. Let $f_{x,y}(\cdot)$ denote the probability density function of a Gamma distribution of shape x and scale y. We can derive the distribution of the number of between-group transmission events conditional on the number of generations as:

$$\begin{aligned}
P[J = j \mid G = g] &= \int_{t=0}^{\infty} P[J = j \mid G = g, T^{evo} = t] \cdot p(t \mid G = g)\, dt \\
&= \int_{t=0}^{\infty} \frac{(\lambda t)^j \cdot e^{\lambda t}}{j!} \cdot f_{\alpha g,\beta}(t) dt \\
&= \int_{t=0}^{\infty} \frac{(\lambda t)^j \cdot e^{\lambda t}}{j!} \cdot \frac{\beta^{\alpha g} \cdot t^{\alpha g - 1} \cdot e^{-\beta t}}{\Gamma(\alpha g)} dt \\
&= \frac{\lambda^j \beta^{\alpha g}}{j!\,\Gamma(\alpha g)} \int_{t=0}^{\infty} \frac{\Gamma(j + \alpha g)}{(\lambda + \beta)^{j+\alpha g}} \cdot f_{j+\alpha g, \lambda+\beta}(t)\, dt \\
&= \frac{\Gamma(j + \alpha g)}{j!\,\Gamma(\alpha g)} \cdot \left(\frac{\beta}{\beta + \lambda}\right)^{\alpha g} \cdot \left(\frac{\lambda}{\beta + \lambda}\right)^{j}
\end{aligned}$$

which is the probability mass function of a negative binomial distribution of parameters $r_{J|g} = \alpha g$ and $p_{J|g} = \frac{\beta}{\beta+\lambda}$.

Therefore:

$$J_{|G=g} \sim NB\left(\alpha g, \frac{\beta}{\beta + \lambda}\right)$$

Distribution of the number of mutations conditional on the number of generations.

By adapting the above demonstration to M, that follows a Poisson process of rate μ, we have:

$$M_{|G=g} \sim NB\left(\alpha g, \frac{\beta}{\beta + \mu}\right)$$

Distribution of the number of between-group transmission events conditional on the number of mutations

We introduce $h(k; r, p)$ as the probability mass function, evaluated in k of a negative binomial distribution of parameters r and p. Then, we have:

$$\begin{aligned}
P[J = j \mid M = m] &= \sum_{g \geq 1} P[J = j \mid G = g, M = m] \cdot P[G = g \mid M = m] \\
&= \sum_{g \geq 1} [J = j \mid G = g] \cdot P[M = m \mid G = g] \cdot \frac{P[G = g]}{P[M = m]} \\
&= \frac{\sum_{g \geq 1} P[J = j \mid G = g] \cdot P[M = m \mid G = g] \cdot P[G = g]}{\sum_{g \geq 1} P[M = m \mid G = g] \cdot P[G = g]}
\end{aligned}$$

$$= \frac{\sum_{g\geq 1} h\left(j; \alpha g, \frac{\beta}{\beta+\lambda}\right) \cdot h\left(m; \alpha g, \frac{\beta}{\beta+\mu}\right) \cdot \pi_g}{\sum_{g\geq 1} h\left(m; \alpha g, \frac{\beta}{\beta+\mu}\right) \cdot \pi_g}$$

Confusion matrix parameters derivation

Sensitivity

For $\Delta \geq 1$, we have:

$$\eta_\Delta = P[\, M \leq \Delta \mid J \geq 1\,]$$
$$= \sum_{d=0}^{\Delta} P[M = \; d \mid J \geq 1]$$

We introduce $\eta_\Delta{}'$ as:

$$\eta'_\Delta = \frac{(P[\, J = 0 \mid M = \Delta\,] + P[\, J = 1 \mid M = \Delta\,]) \cdot P[M = \Delta]}{P[J \geq 1]}$$

Therefore, for all $\Delta \geq 0$, we have:

$$\eta_\Delta = \sum_{d=0}^{\Delta} \eta_d{}'$$

Specificity

For $\Delta \geq 1$, we have:

$$\chi_\Delta = P[\, M > \Delta \mid J > 1\,]$$
$$= 1 - \mathrm{P}[\, \mathrm{M} \leq \Delta \mid J > 1\,]$$
$$= 1 - \sum_{d=0}^{\Delta} P[M = d \mid J > 1]$$

For $\Delta \geq 0$, we introduce:

$$\chi'_\Delta = \frac{(1 - P[J = 0 \mid M = \Delta] - P[J = 1 \mid M = \Delta]) \cdot P[M = \Delta]}{1 - P[J = 0] - P[J = 1]}$$

Therefore, for all $\Delta \geq 0$, we have:

$$\chi_\Delta = 1 - \sum_{d=0}^{\Delta} \chi_d{}'$$

Characteristics of spatial and age-based transmission processes.

Age mixing from social contact data. We explore the probability for transmission to occur within the same age group using synthetic social contact data for Washington state from Mistry et al[2]. The latter study provides estimates of the mean daily number of contacts $M_{i,j}$ that individuals of age i have with individuals of age j (with one-year age bins). Let n_i denote the number of individuals of age i. Age groups can be defined by specifying an aggregation rule. The total number of contacts $\Gamma_{A,B}$ that occur within one day between two population groups A and B follows:

$$\Gamma_{A,B} = \sum_{i \in A} \sum_{j \in B} M_{i,j} \cdot n_i$$

We can also define $c_{A,B}$ the average daily number of contacts that individuals within age group *A* have with individuals in age group *B* as:

$$c_{A,B} = \frac{\Gamma_{A,B}}{\sum_{i \in A} n_i}$$

We compute the proportion $p^{within\ age}$ of contacts occurring within the same age groups across all contacts occurring within one day as:

$$p^{within\ age} = \frac{\sum_A \Gamma_{A,A}}{\frac{1}{2}\sum_A (\sum_{B \neq A} \Gamma_{A,B}) + \sum_A \Gamma_{A,A}}$$

The normalizing factor ½ is used to ensure each contact is only counted once. This metric is a summary statistic of within-group transmission probability at the population level for a specified level of age aggregation. In practice, the probability for a contact of occurring within the same age group is not constant across age groups (Figure S1).

We compute values of $p^{within\ age}$ for different binning window size: 1-year, 2-year, 5-year, 10-year and 20-year age bins (Figure S2). For each binning scenario, aggregation starts from age 0 and stops at age 79. We systematically include an age group corresponding to individuals aged 80 and older.

Spatial mixing from mobility data. We estimate the probability for transmission of occurring within the same geographical region using mobile device location data from SafeGraph (https://safegraph.com/), a data company that aggregated anonymized location data from 40 million devices, or approximately 10% of the US population, to over 6 million physical places (points of interest, POIs). We use the probability for a movement of occurring within the same geographical unit, while exploring different scales in the US: states, counties, Public Use Microdata Areas (PUMAs), census tracts, and census block groups (CBGs).

At the state and county level, we use data processed in Pullano et al.[3] and made publicly available in the associated GitHub repository[4]. The authors report the proportion $p_{i,j}$ of movements of people living in county i that travel to county j, across different states and for a range of time windows. We focus here on data from January 2020. To estimate the proportion of movements that occur within the same county, we compute a population-weighted average of the proportion of movements occurring within the same county as follows:

$$p^{within\ county} = \frac{\sum_i p_{i,i} \cdot N_i}{\sum_i N_i}$$

where N_i is the population size of county i, derived from US Census data[5]. Using a similar definition, we estimate the proportion $p^{within\ state}$ of movements that occur within the same state.

For smaller geographical units (PUMAs, census tracts and CBGs), we rely on a Washington state (WA) focused dataset[6]. We use SafeGraph's Weekly Patterns dataset to estimate movements within and between WA geographies between January 2019 and June 2022. This dataset provides weekly counts of the total number of unique devices visiting a POI from a particular home location. We restrict our analysis to POIs that are consistently recorded in SafeGraph's panel throughout the study period.

To measure movement within and between CBGs, we extract the home CBG of devices visiting POIs and limited the dataset to devices with home locations in the CBG of a given POI (within-CBG movement) or with home locations in CBGs outside of a given POI's CBG (between-CBG movement). This methodology was also applied to census tracts and PUMAs to measure movement within and between these larger geographic units.

To adjust for variation in the size of SafeGraph device panel over time, we multiply raw weekly visits to POIs by a scaling factor, corresponding to the monthly ratio of each CBG, census tract or PUMA's respective county census population size to the number of devices in SafeGraph's panel with home locations within that county. We then compute the total number of visits between geographies by summing adjusted weekly counts across POIs, over the entire study period. We use these adjusted counts to compute the proportion of movements occurring within the same county, PUMA, census tract and CBG in WA. Estimated values for the proportion of movements within each geographical unit are detailed in Table S1.

Simulation study to explore the relationship between power and sample size

We evaluate how sample size influences the ability to characterize transmission patterns between groups from sequence data by performing a simulation study using the ReMASTER BEAST2 package[7].

Simulations parametrization

We modelled SEIR epidemics characterized by a basic reproduction number of 2, with a rate out of the exposed (E) compartment of 0.33/day and a rate out of the infected (I) compartment of 0.33/day. This corresponds to a Gamma distributed generation time with shape $\alpha = 2$ and scale $\beta = 1/0.33$ day. We consider the spread of a pathogen characterized by a probability p that transmission occurs before mutation (exploring values between 0.1 and 0.9 with an increment of 0.1) between 4 population groups each of size 50,000. This probability p and the generation time's parametrization as a Gamma distribution translates to a per genome mutation rate of:

$$\mu^{per\ genome} = \beta(p^{-\frac{1}{\alpha}} - 1)$$

by using similar arguments to those in subsection *Relationship between the probability for the infectee to be in the same subgroup as the infector and the between-group transmission event rate λ*. Assuming a Jukes-Cantor model of evolution, we derive the per site mutation rate (which is used in the simulations) as:

$$\mu^{per\ site} = \frac{1}{l} \cdot \mu^{per\ genome}$$

where l is the genome length. We run simulations assuming a genome length of 3,000 bp.

We consider transmission processes characterized by within-group transmission probabilities ω ranging between 0.1 and 0.9 with an increment of 0.1. We assume a symmetric mixing matrix between groups (detailed parametrization in Table S2).

We assume that a fraction p_{seq} of all infections are sequenced (exploring values of 0.001, 0.005, 0.01 and 0.05).

Relative risk metric performance

To assess the ability of sequences below a given genetic distance threshold to capture mixing patterns, we compute a relative risk (RR) metric which was introduced in prior work and that was shown to capture SARS-CoV-2 transmission patterns between age groups and geographies[1].

Let $H_{i,j}$ denote the Hamming distance separating two sequences indexed i and j, let S_i denote the population subgroup to which sequence i belongs. Let n denote the number of sequences in the dataset. We define the relative risk $RR_{A,B}^{\Delta}$ of observing two sequences less than Δ mutations away in population groups A and B as:

$$RR_{A,B}^{\Delta} = \frac{n_{A,B}^{\Delta} \cdot n_{\bullet,\bullet}^{\Delta}}{n_{A,\bullet}^{\Delta} \cdot n_{B,\bullet}^{\Delta}}$$

where (using $\mathbf{1}$ to denote the indicator function):

$$n_{A,B}^{\Delta} = \sum_{i=1}^{n}\sum_{j=1}^{n} \mathbf{1}_{\{H_{i,j} \le \Delta\}} \cdot \mathbf{1}_{\{i \ne j\}} \cdot \mathbf{1}_{\{S_i = A\}} \cdot \mathbf{1}_{\{S_j = B\}}$$

$$n_{A,\bullet}^{\Delta} = \sum\nolimits_{B} n_{A,B}^{\Delta}$$

$$n_{\bullet,\bullet}^{\Delta} = \sum\nolimits_{A}\sum\nolimits_{B} n_{A,B}^{\Delta}$$

In situations where there aren't any pairs of sequences observed in either subgroup A or subgroup B ($n_{A,\bullet}^{\Delta}$ or $n_{B,\bullet}^{\Delta}$ is equal to 0), $RR_{A,B}^{\Delta}$ is not defined. To compute RRs even in situations of low pair counts, we rely on a modified RR, defined as:

$$\widetilde{RR}_{A,B}^{\Delta} = \frac{(n_{A,B}^{\Delta} + 1) \cdot (n_{\bullet,\bullet}^{\Delta} + 1)}{(n_{A,\bullet}^{\Delta} + 1) \cdot (n_{B,\bullet}^{\Delta} + 1)}$$

For each combination of p_{seq}, ω and p, we simulate 50 outbreaks with associated sequence data and compute modified relative risks ($\widetilde{RR}$) for thresholds Δ ranging between 0 and 15. We then compute the Spearman correlation coefficient between RRs and daily between-group transmission probabilities. In simulations where the modified RRs' standard deviation is equal to 0, we set the correlation coefficient to 0 (RRs are not informative about between-group transmission probabilities). For each combination of p_{seq}, ω, p and Δ, we compute the median correlation across the 50 replicate simulations $\rho^{50}(p_{seq}, \omega, p, \Delta)$. To characterize the best inference performance for a given sequencing effort p_{seq}, we compute the maximum median correlation across Δ ranging between 0 and 15:

$$\rho^{50,max}(p_{seq}, \omega, p) = \max_{0 \le \Delta \le 15} \rho^{50}(p_{seq}, \omega, p, \Delta)$$

We then characterize the minimum level of sequencing effort required to reach a correlation threshold τ (50% and 90%) for each combination of ω and p as:

$$p_{seq}^{required\ \tau}(\omega, p) = \min_{p_{seq} \in \{0.001, 0.005, 0.01, 0.05\}} \{p_{seq} \mid \rho^{50,max}(p_{seq}, \omega, p) \ge \tau\}$$

Supplementary tables

Table S1: Estimates of the probability for a movement of occurring within the same geographical unit in the US.

Geographical unit	Data source	Proportion
Census block groups	SafeGraph in Washington state	0.05
Census tracts	SafeGraph in Washington state	0.10
Public Use Microdata Areas (PUMAs)	SafeGraph in Washington state	0.52
Counties	SafeGraph in Washington state	0.81
Counties	Safegraph data from Pullano et al.	0.76
States	Safegraph data from Pullano et al.	0.90

Table S2: Mixing matrix used in the ReMASTER simulations as a function of the within-group transmission probability parameter ω used in the parametrization. Each coefficient corresponds to the probability that an infection coming from someone belonging to group i (rows) is in someone in group j (columns).

ω	$(1-\omega)\cdot 1/12$	$(1-\omega)\cdot 4/12$	$(1-\omega)\cdot 7/12$
$(1-\omega)\cdot 1/12$	ω	$(1-\omega)\cdot 7/12$	$(1-\omega)\cdot 4/12$
$(1-\omega)\cdot 4/12$	$(1-\omega)\cdot 7/12$	ω	$(1-\omega)\cdot 1/12$
$(1-\omega)\cdot 7/12$	$(1-\omega)\cdot 4/12$	$(1-\omega)\cdot 1/12$	ω

Supplementary figures

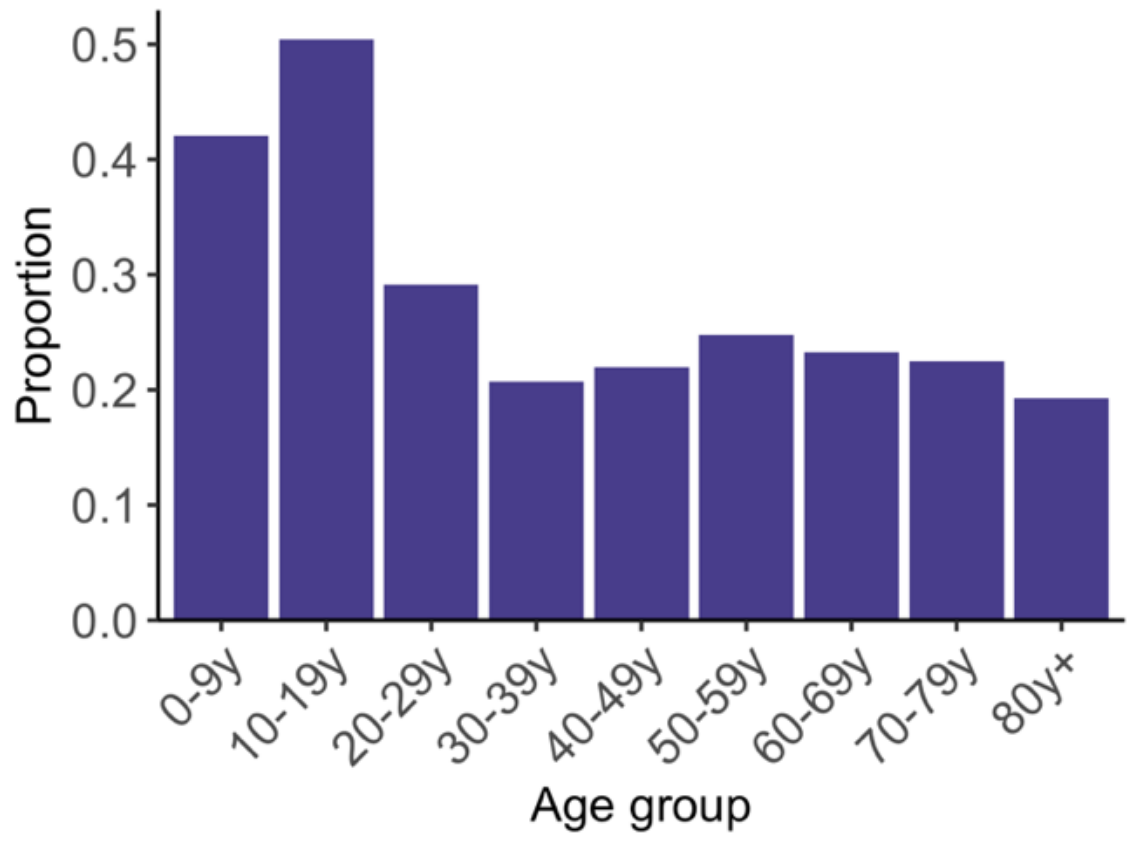

Figure S1: Proportion of contacts occurring within the same age group across age groups, where age group are defined in decades. Estimates were obtained using synthetic social contact data from Washington state[2].

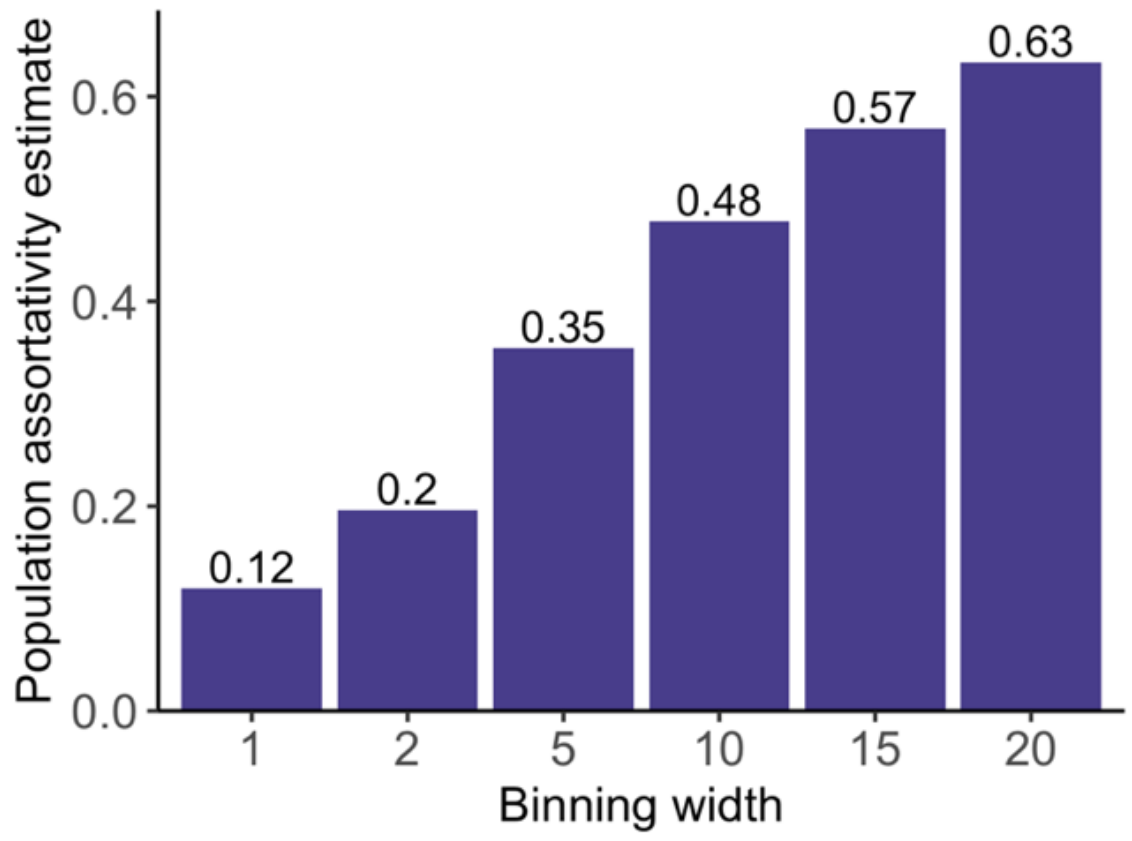

Figure S2: Estimates of the probability for a contact of occurring within the same age group as a function of the binning window width used to define age groups. Estimates were obtained using synthetic social contact data from Washington state[2].

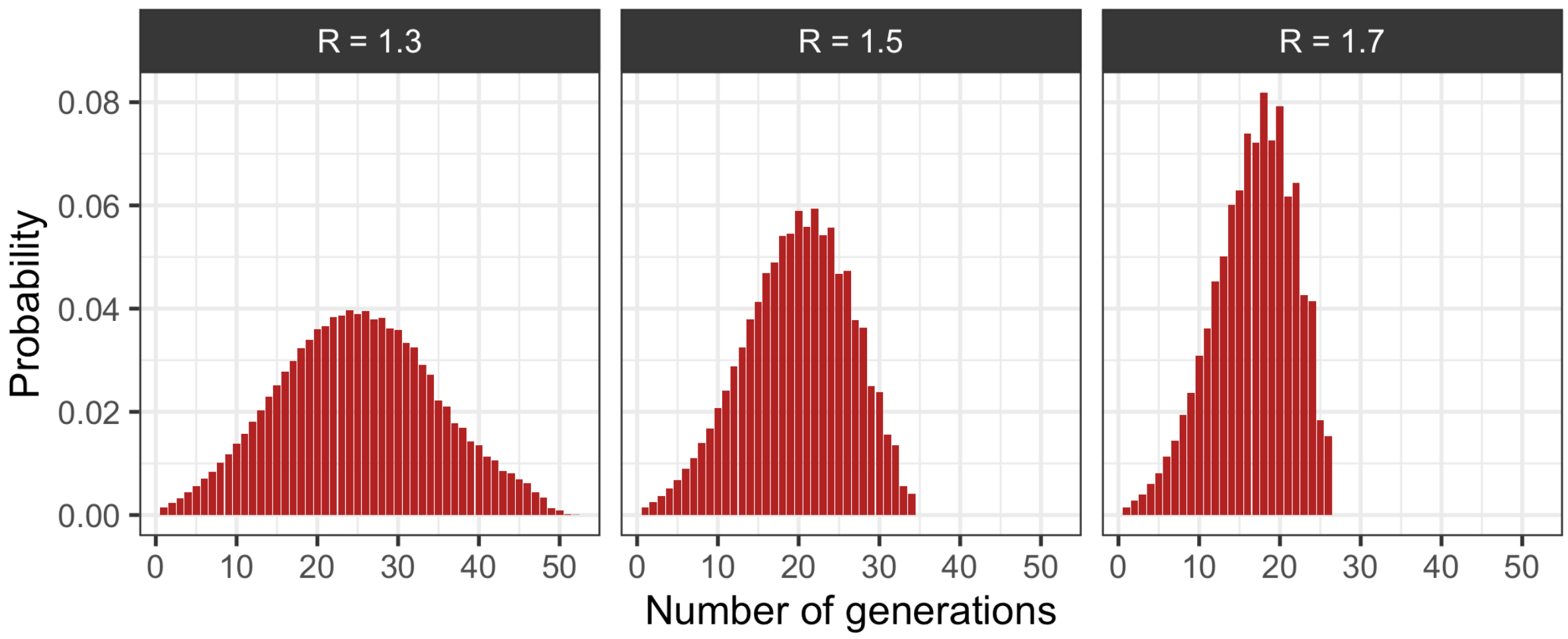

Figure S3: Distribution of the number of generations separating two infected individuals used in the computations. These probabilities are directly extracted from the *phylosamp* R package[8] as estimated by Wohl et al[9].

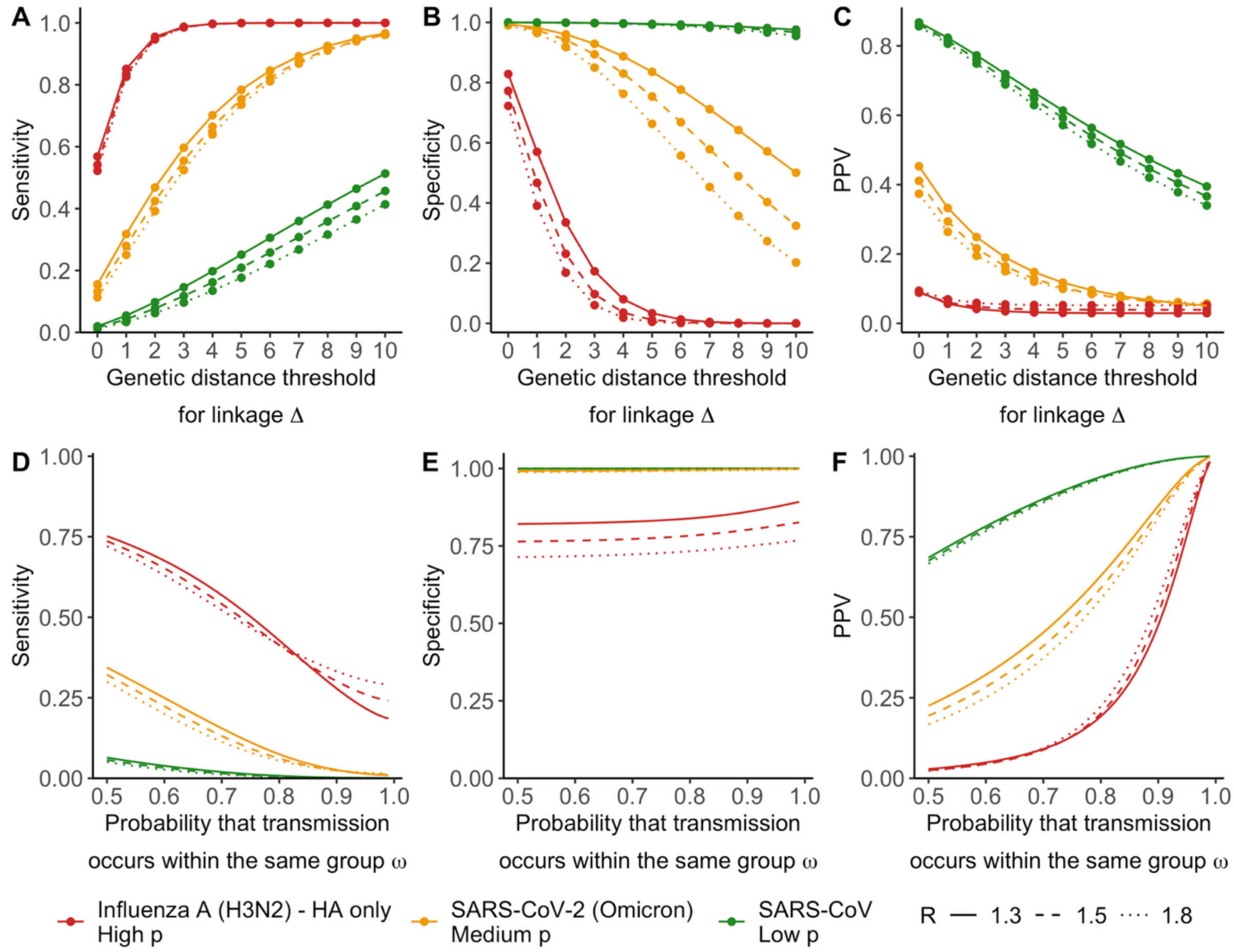

Figure S4: Sensitivity analysis exploring sensitivity, specificity and PPV for different values for the reproduction number R. A. Sensitivity, **B.** specificity and **C.** PPV as a function of the genetic distance threshold used to define the linkage criterion and assuming a within-group transmission probability ω of 0.7. **D.** Sensitivity, **E.** specificity and **F.** PPV of a linkage criterion defined by $\Delta = 0$ as a function of the within-group transmission probability ω.

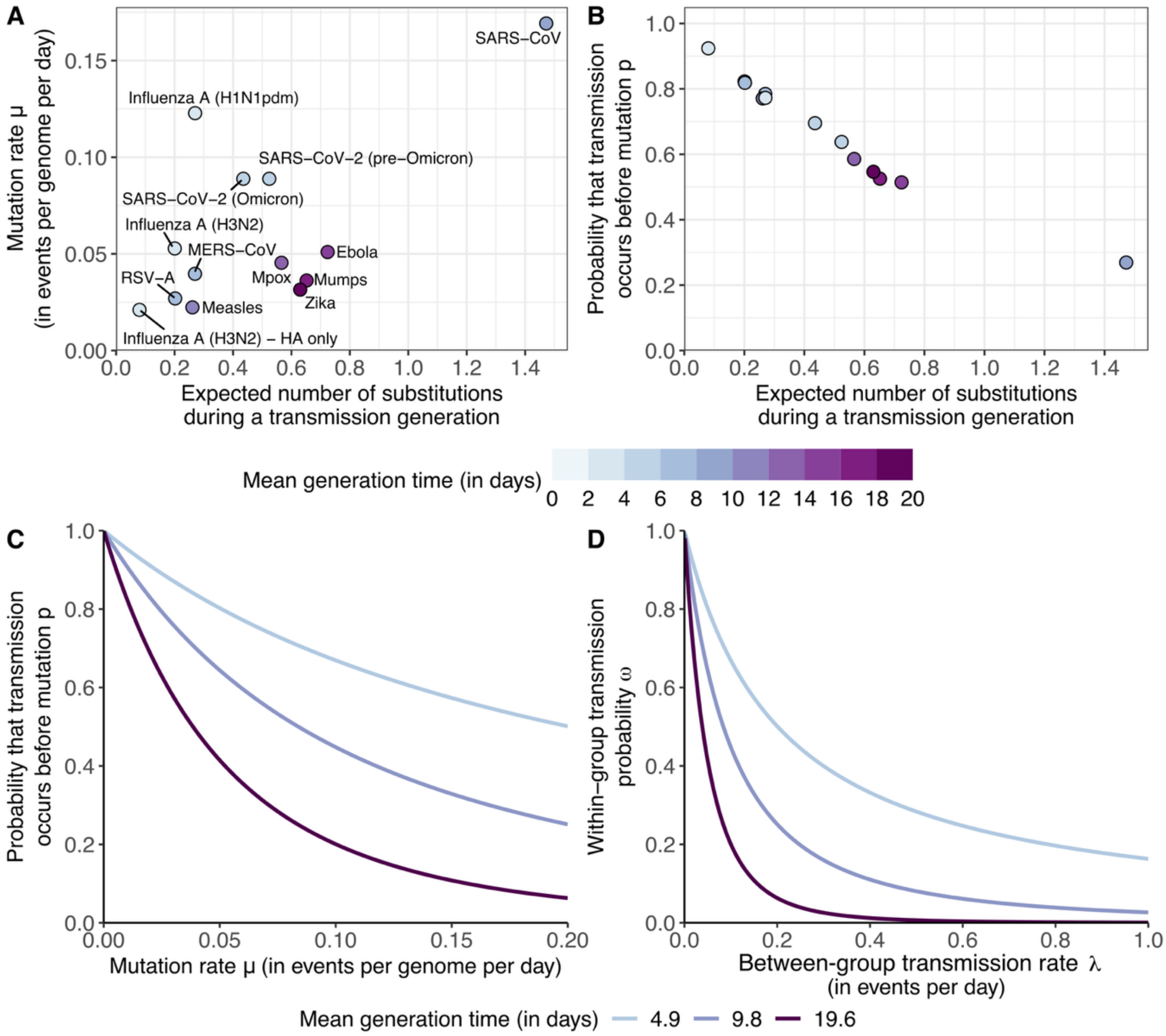

Figure S5: Rescaling pathogen's evolutionary rates and between-group transmission rates accounting for generation time distribution. A. Relationship between the mutation rate μ and the expected number of substitutions during a transmission generation across a range of pathogens. **B.** Relationship between the probability that transmission occurs before mutation p and the expected number of substitutions during a transmission generation. **C.** Relationship between μ and p for different generation time distribution parametrizations. **D.** Relationship between the between-group transmission rate λ and the within-group transmission probability ω for different generation time distribution parametrizations. In C and D, we considered Gamma distributed generation time with the same scale as the one estimated for SARS-CoV-2 (0.21 – see Methods).

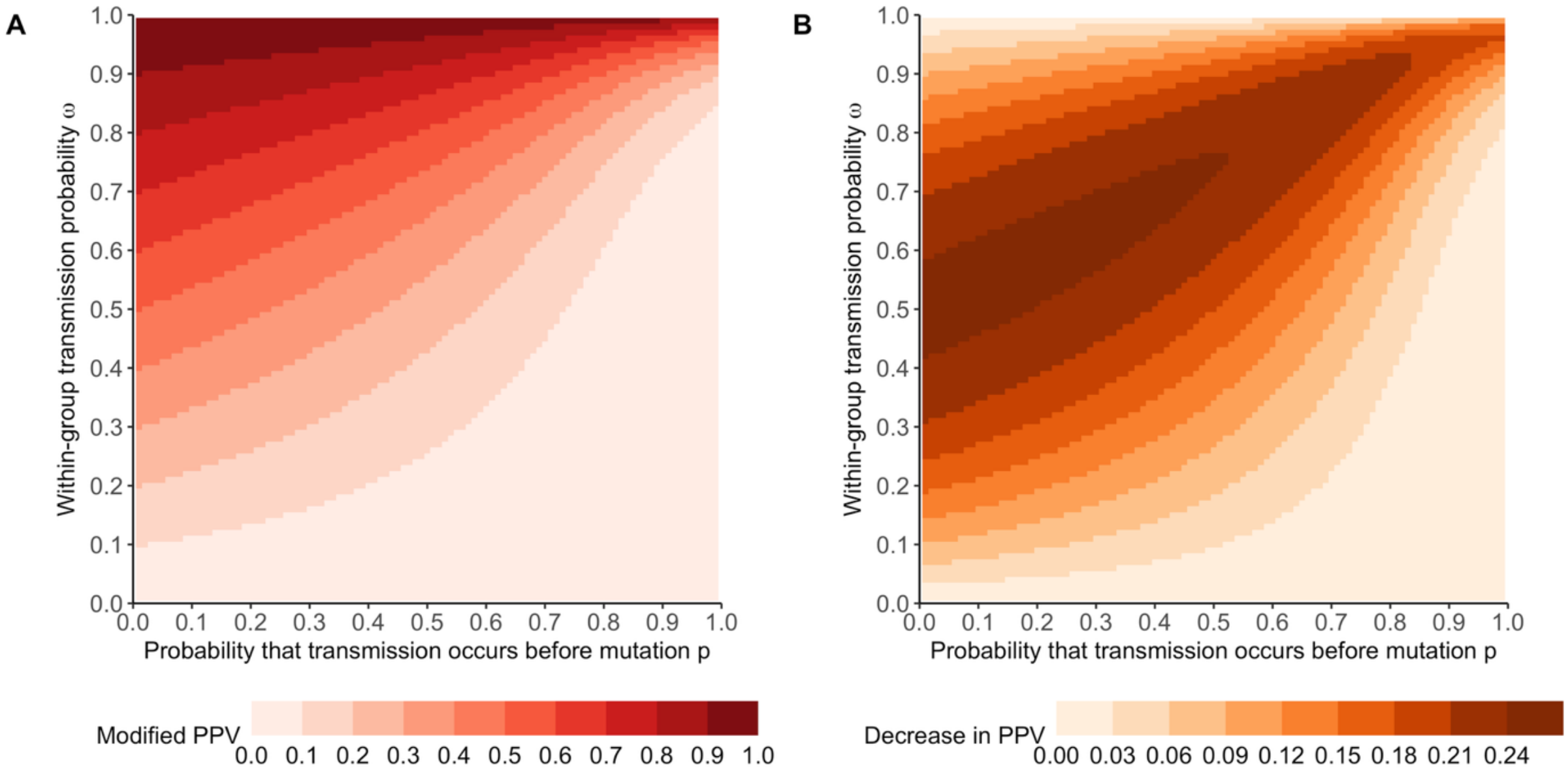

Figure S6: Impact of classifying pairs of sequences characterized by $J = 0$ as True Negatives on the PPV. A. Modified PPV as a function of the probability that transmission occurs before mutation p and the within-group transmission probability ω. **B.** Decrease in the PPV when classifying pairs of sequences characterized by $J = 0$ as TN instead of TP. The modified PPV $\widetilde{\phi_\Delta}$ was computed as:

$$\widetilde{\phi_\Delta} = P[J = 1 \mid M \leq \Delta, J \geq 1] = \frac{\phi_\Delta - P[J = 0 \mid M \leq \Delta]}{1 - [J = 0 \mid M \leq \Delta]}$$

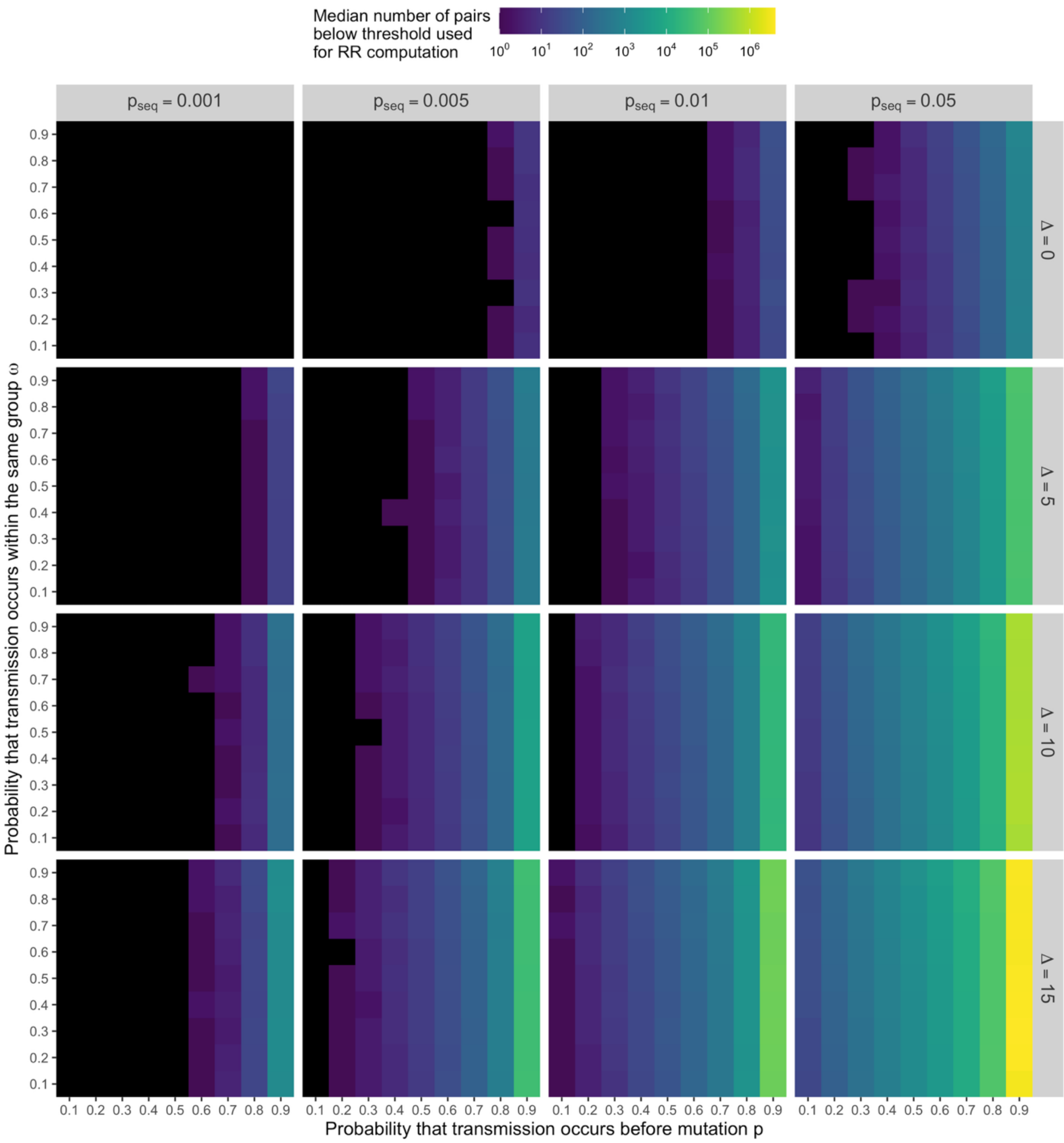

Figure S7: Median number of pairs of sequences less than Δ mutations away across 50 replicate simulations as a function exploring different sequencing fractions p_{seq} and genetic distance thresholds Δ. Results are displayed as a function of p and ω. Black tiles correspond to a median value of 0.

References

1. Tran-Kiem, C. *et al.* Fine-scale patterns of SARS-CoV-2 spread from identical pathogen sequences. *Nature* (2025) doi:10.1038/s41586-025-08637-4.
2. Mistry, D. *et al.* Inferring high-resolution human mixing patterns for disease modeling. *Nat. Commun.* **12**, 323 (2021).
3. Pullano, G., Alvarez-Zuzek, L. G., Colizza, V. & Bansal, S. Characterizing US spatial connectivity and implications for geographical disease dynamics and metapopulation modeling: Longitudinal observational study. *JMIR Public Health Surveill.* **11**, e64914 (2025).
4. Pullano, G. *US-Connectivity-Metapop*. (GitHub, 2025).
5. Reinhart, A. *et al.* An open repository of real-time COVID-19 indicators. *Proc. Natl. Acad. Sci. U. S. A.* **118**, e2111452118 (2021).
6. Perofsky, A. C. *et al.* Impacts of human mobility on the citywide transmission dynamics of 18 respiratory viruses in pre- and post-COVID-19 pandemic years. *Nat. Commun.* **15**, 4164 (2024).
7. Vaughan, T. G. ReMASTER: improved phylodynamic simulation for BEAST 2.7. *Bioinformatics* **40**, (2024).
8. Lee, E., Wohl, S., Giles, J. & McGowan, L. D. *HopkinsIDD/Phylosamp: Phylosamp v1.0.1 (CRAN Release)*. (Zenodo, 2023). doi:10.5281/ZENODO.7964186.
9. Wohl, S., Giles, J. R. & Lessler, J. Sample size calculation for phylogenetic case linkage. *PLoS Comput. Biol.* **17**, e1009182 (2021).
